# Supplementary material for: Structural reconstruction of individual filaments in Aβ42 fibril populations assembled in vitro reveal rare species that resemble ex vivo amyloid polymorphs from human brains
Source: Commun Chem. 2025 Nov 19;8:358. doi: 10.1038/s42004-025-01741-6 (PMC12630867; doi:10.1038/s42004-025-01741-6)
Supplement: Supplementary file 2 — Supplementary Information [file 42004_2025_1741_MOESM2_ESM.pdf]

Structural reconstruction of individual filaments in $A\beta_{42}$ fibril populations assembled *in vitro* reveal rare species that resemble *ex vivo* amyloid polymorphs from human brains

Supplementary Information

Liam D. Aubrey[1,3], Liisa Lutter[1,4], Kate Fennell[2], Tracey J. Purton[1], Natasha L. Ward[1], Louise C. Serpell[2], Wei-Feng Xue[1]*

[1] School of Natural Sciences, University of Kent, CT2 7NJ, Canterbury, UK

[2] Sussex Neuroscience, School of Life Sciences, University of Sussex, BN1 6NN, Falmer, Brighton, UK

[3] Current address: Astbury Centre for Structural Molecular Biology, School of Molecular and Cellular Biology, Faculty of Biological Science, University of Leeds, Leeds, LS2 9JT, UK

[4] Current address: Molecular Biology Institute, UCLA, Los Angeles, CA 90095, USA

* Correspondence to: W.F.Xue@kent.ac.uk; Tel +44-(0)1227 824821

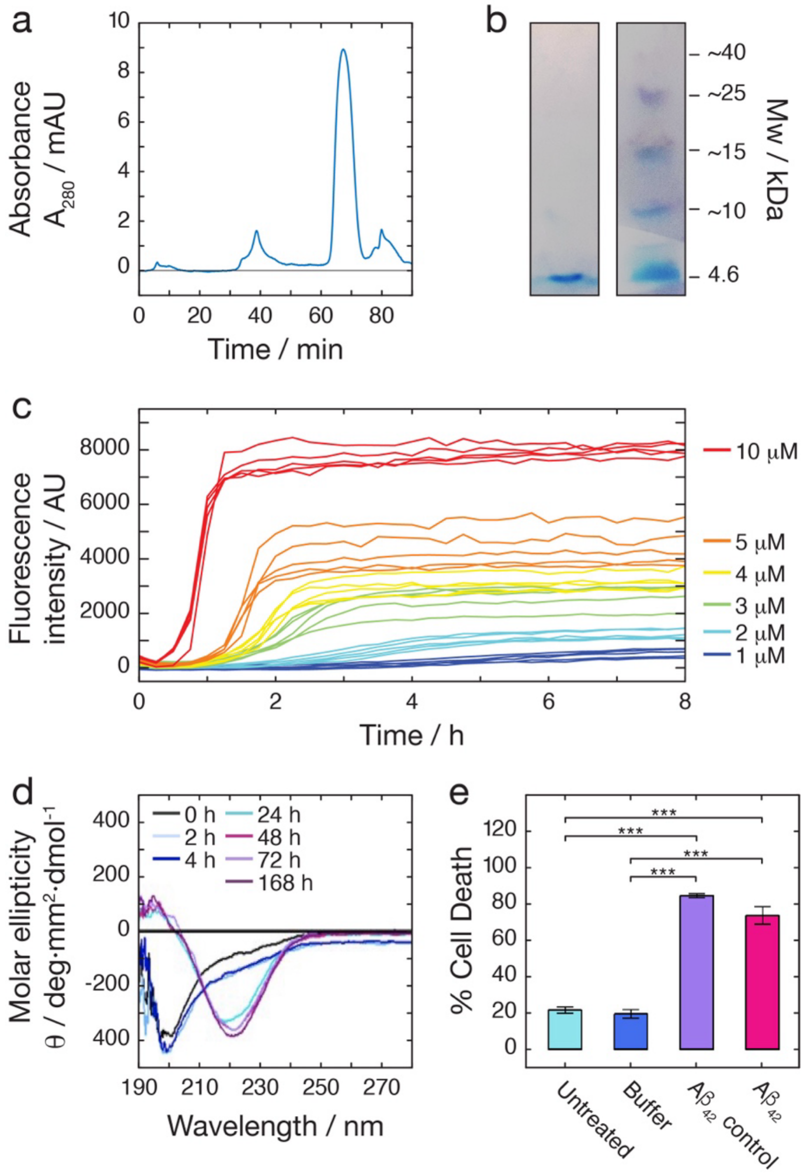

Supplementary Figure S1: Expression and purification of Aβ42. (a) Size exclusion chromatography trace of monomeric recombinant Aβ42 elution immediately before use. Aβ42 was expressed and purified as described in the Methods section, with the last purification step consisting of multiple rounds of SEC, which were performed until only a single peak appeared on the chromatogram. (b) SDS-PAGE confirming the purified Aβ42 monomer sample used. Fibril formation was tracked by ThT assay (c) and by CD (d), demonstrating the expected assembly competency of the monomeric recombinant Aβ42 samples. (e) The cytotoxicity of

small oligomeric species formed from the monomeric recombinant Aβ_{42} sample compared to species formed using a commercial sample (Aβ_{42} control) from rPeptide [39] demonstrate the expected cytotoxic potential for the recombinant Aβ_{42} samples.

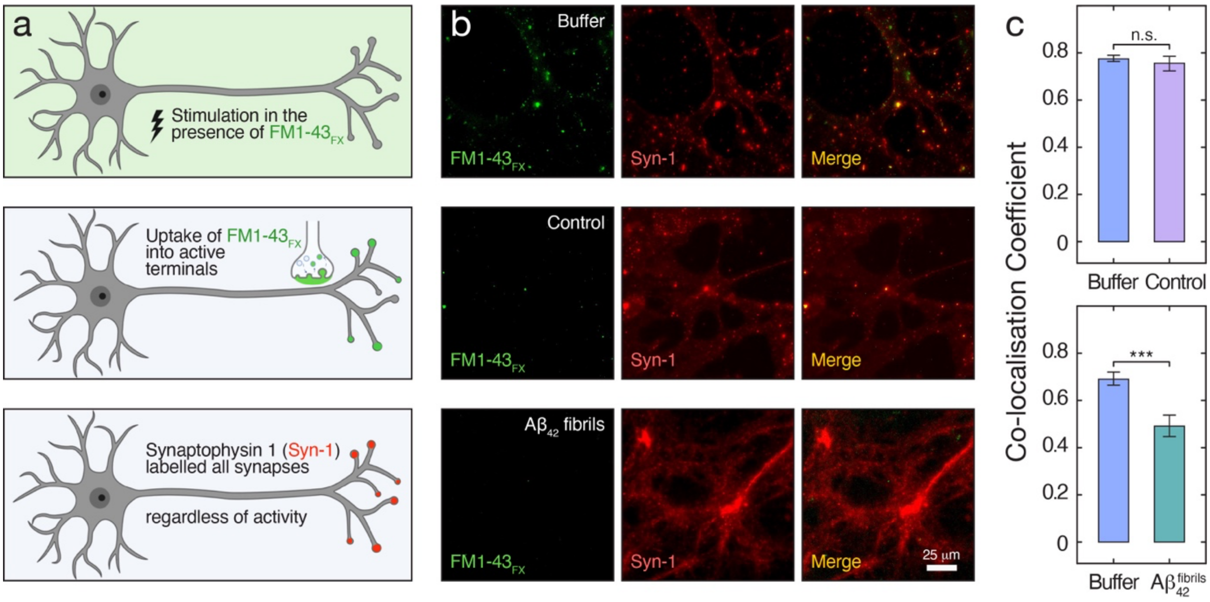

Supplementary Figure S2: Aβ42 fibrils formed *in vitro* demonstrate detrimental effects on neuronal function. (a) Schematic illustration of the experimental assay of synaptic function. Primary mice hippocampal neurons were subjected to a 600 AP stimulation in the presence of FM1-43FX, which labelled functional synaptic terminals. Co-staining with anti-synaptophysin 1 (Syn-1) labelled all synapses regardless of activity. (b). Images showing a 24-hour treatment with either buffer, assembly-incompetent Aβ42 control ($A\beta^{Var}{}_{42}$) which did not significantly alter the number of active terminals, or fibrillar Aβ42 which significantly reduced the number of functional synapses. Images of separate FM1-43FX and Syn-1 channels, and a composite image containing both channels are shown with the scale bar indicating 25 μm in all images. (c) Comparison of synaptic function quantified by Mander's co-localisation coefficient M1 indicate that the pathogenic nature of Aβ42 is dependent on its ability to assemble from monomers and oligomers into fibrils. Quantification for each condition shown is from three independent replicates with the error bars indicate standard error of mean and *** indicate significant difference of means determined using one-way ANOVA and Tukey's test with a p-value < 0.001.

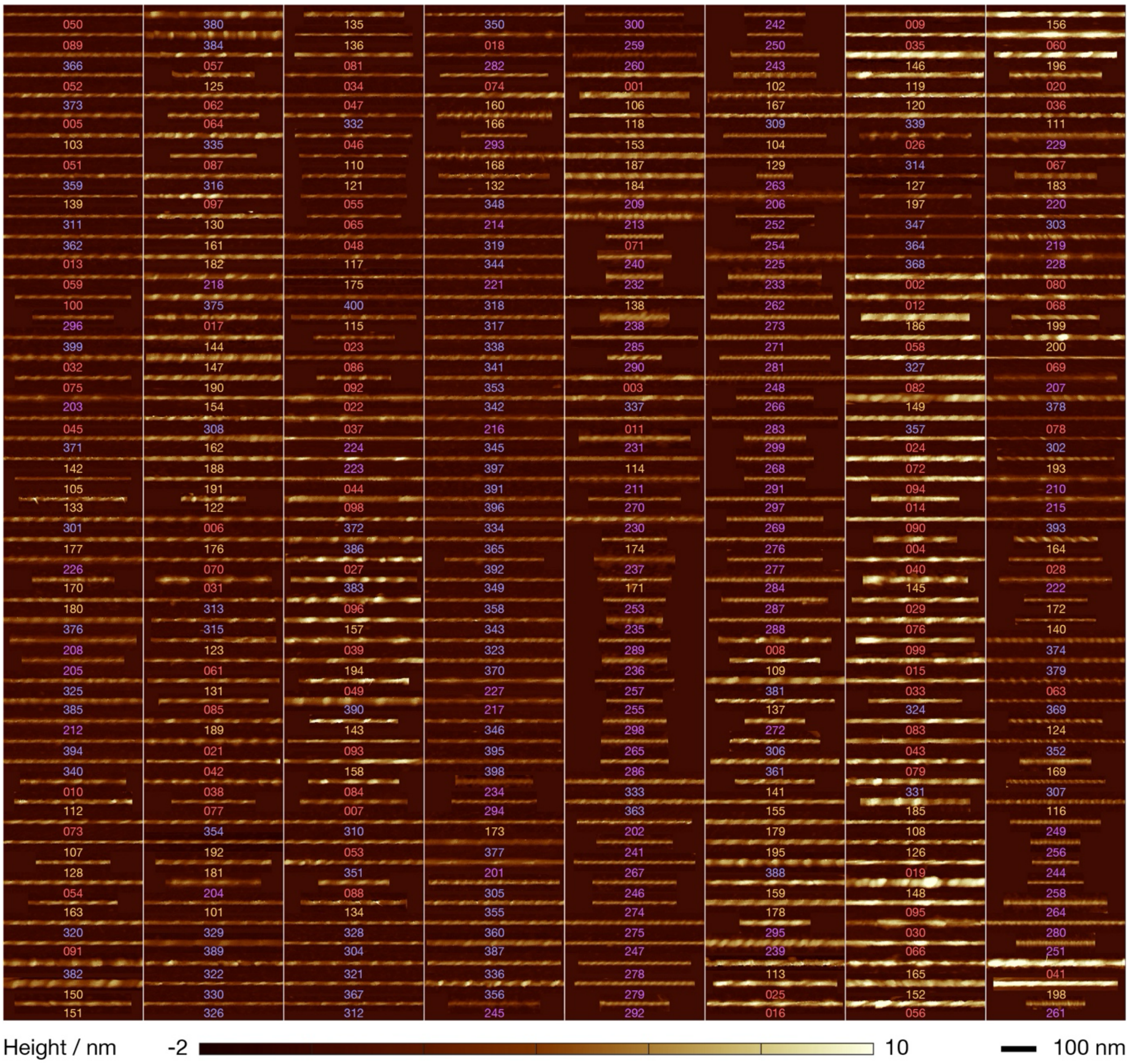

Supplementary Figure S3: AFM image data for all of the 400 individually analysed Aβ_{42}. fibrils. Up to a 400 nm of digitally straightened segment are shown for each individual fibril analysed. The fibrils are shown with their individual index numbers (see **Supplementary Table 2**) coloured coded according to the assembly condition they were found in (20mM of sodium phosphate pH 8: red #1-100, sodium phosphate pH 7.4: orange #101-200, Tris pH 7.4: purple #201-300, and HEPES pH 7.4: blue #301-400). The fibrils are arranged by similarity, in the same order from upper-left column-wise to lower-right as shown from bottom to top of

the dendrogram in Figure 4. All fibril images are identically scaled for comparison, with the colour scale of the images represent height range of -2 to 10 nm as indicated by the bottom colour bar and length scale of 100 nm indicated by the scalebar.

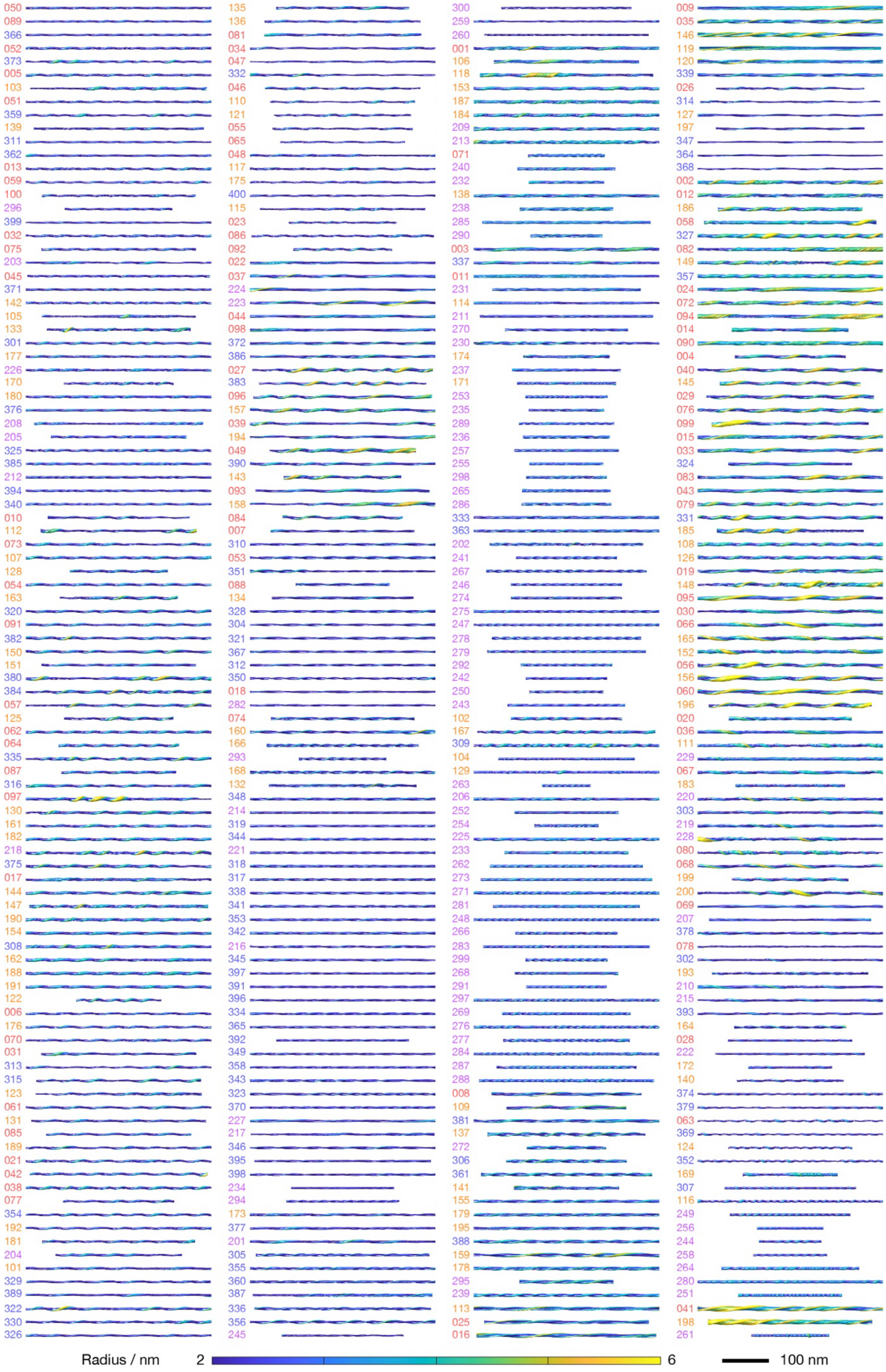

050
089
366
052
373
005
103
051
359
139
311
362
013
059
100
296
399
032
075
203
045
371
142
105
133
301
177
226
170
180
376
208
205
325
385
212
394
340
010
112
073
107
128
054
163
320
091
382
150
151
380
384
057
125
062
064
335
087
316
097
130
161
182
218
375
017
144
147
190
154
308
162
188
191
122
006
176
070
031
313
315
123
061
131
085
189
021
042
038
077
354
192
181
204
101
329
389
322
330
326
135
136
081
034
047
332
046
110
121
055
065
048
117
175
400
115
023
086
092
022
037
224
223
044
098
372
386
027
383
096
157
039
194
049
390
143
093
158
084
007
310
053
351
088
134
328
304
321
367
312
350
018
282
074
160
166
293
168
132
348
214
319
344
221
318
317
338
341
353
342
216
345
397
391
396
334
365
392
349
358
343
323
370
227
217
346
395
398
234
294
173
377
201
305
355
360
387
336
356
245
300
259
260
001
106
118
153
187
184
209
213
071
240
232
138
238
285
290
003
337
011
231
114
211
270
230
174
237
171
253
235
289
236
257
255
298
265
286
333
363
202
241
267
246
274
275
247
278
279
292
242
250
243
102
167
309
104
129
263
206
252
254
225
233
262
273
271
281
248
266
283
299
268
291
297
269
276
277
284
287
288
008
109
381
137
272
306
361
141
155
179
195
388
159
178
295
239
113
025
016
009
035
146
119
120
339
026
314
127
197
347
364
368
002
012
186
058
327
082
149
357
024
072
094
014
090
004
040
145
029
076
099
015
033
324
083
043
079
331
185
108
126
019
148
095
030
066
165
152
056
156
060
196
020
036
111
229
067
183
220
303
219
228
080
068
199
200
069
207
378
078
302
193
210
215
393
164
028
222
172
140
374
379
063
369
124
352
169
307
116
249
256
244
258
264
280
251
041
198
261
Radius / nm
2
6
100 nm

Supplementary Figure S4: 3D models of all of the 400 individually analysed Aβ_{42}. fibrils. Up to a 400 nm of reconstructed fibril 3D surface envelopes are displayed for each individual fibril analysed. The fibrils are shown with their individual index numbers (see **Supplementary Table 2**) coloured coded according to the assembly condition they were found in (20mM of sodium phosphate pH 8: red #1-100, sodium phosphate pH 7.4: orange #101-200, Tris pH 7.4: purple #201-300, and HEPES pH 7.4: blue #301-400). The fibrils are arranged by similarity, in the identical order from upper-left column-wise to lower-right as shown in Supplementary Figure S2 and from bottom to top of the dendrogram in Figure 4. All of the models are shown with identical scales for comparison, with the colour represent the local radius to the screw axis according to the bottom colour bar for visualisation and length scale of 100 nm indicated by the scalebar.

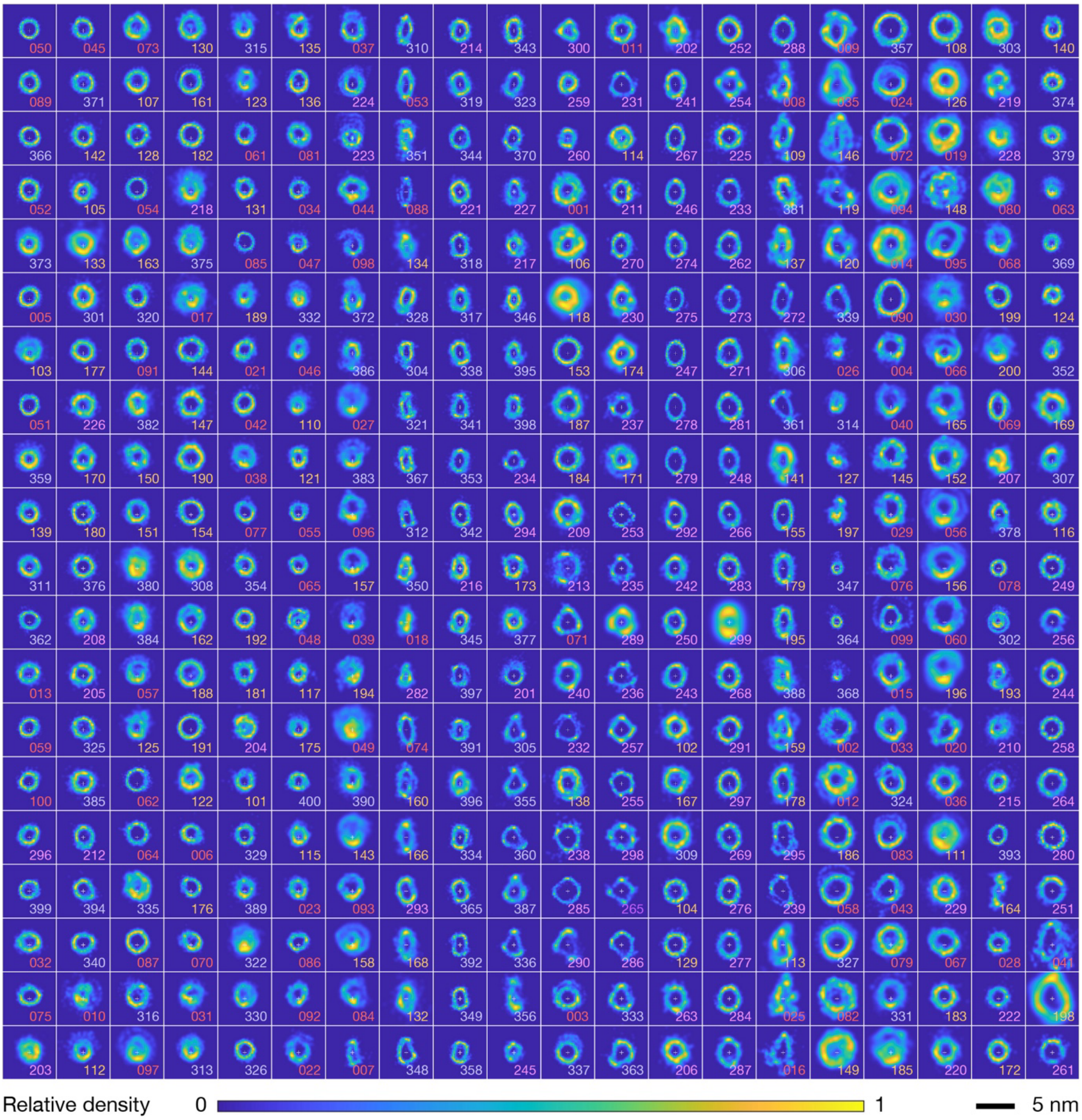

Supplementary Figure S5: Cross-sectional contact-point density map for all of the 400 individually analysed Aβ42. fibrils. The cross-sectional contact-point densities are scaled between 0 to 1 relative to the highest density for each density map. The fibrils are shown with their individual index numbers (see **Supplementary Table 2**) coloured coded according to the assembly condition they were found in (20mM of sodium phosphate pH 8: red #1-100, sodium phosphate pH 7.4: orange #101-200, Tris pH 7.4: purple #201-300, and HEPES pH 7.4: blue

#301-400). The fibrils are arranged by similarity, in the identical order from upper-left column-wise to lower-right as shown in Supplementary Figure S2 and S3, and from bottom to top of the dendrogram in Figure 4. All of the density maps are shown with identical scales for comparison, with the colour represent the relative density and length scale of 5 nm indicated by the scalebar.

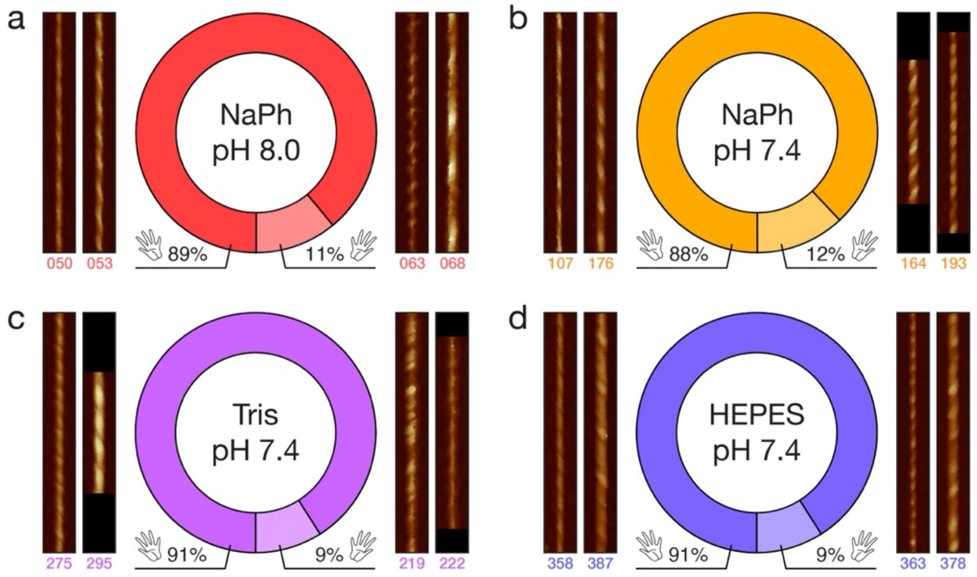

Supplementary Figure S6: Left-hand twisted fibrils represent the majority of fibril populations observed across all four assembly conditions. The proportions of left-hand twisted fibrils versus right-hand twisted fibrils in Aβ_{42} fibril samples formed in (a) 20mM sodium phosphate (NaPh) pH 8.0, (b) sodium phosphate pH 7.4, (c) Tris pH 7.4, and (d) HEPES pH 7.4, are shown together with example fibril images for comparison of their helical twist. Up to a 400 nm for each individual fibril example are shown with their individual index numbers (see **Supplementary Figure S3 and Supplementary Table 2**) coloured coded according to the assembly condition they were found in.

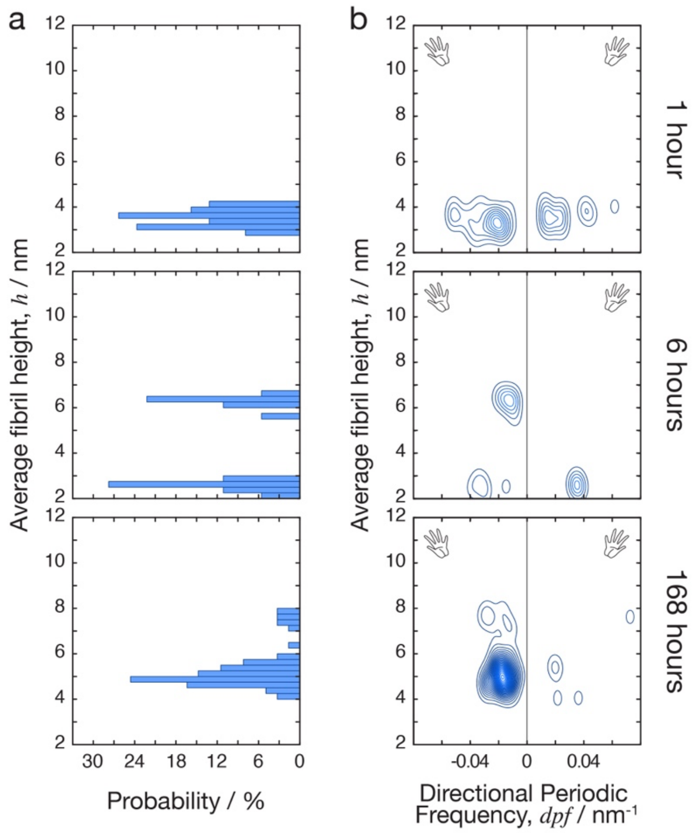

Supplementary Figure S7: The distribution of structural polymorphs of Aβ42 fibrils assemble via intermediate polymorph distribution. Morphometric analysis of Aβ42 fibril assembly in 20mM HEPES pH 7.4 at different time-points. (a) The distribution of average fibril height for the heterogeneous fibril populations formed in each of the assembly time-points monitores. (b) Contour maps displaying average height of individual fibrils (describing their cross-sectional width) plotted versus their individual *dpf* values (describing their helical properties of handedness and pitch).

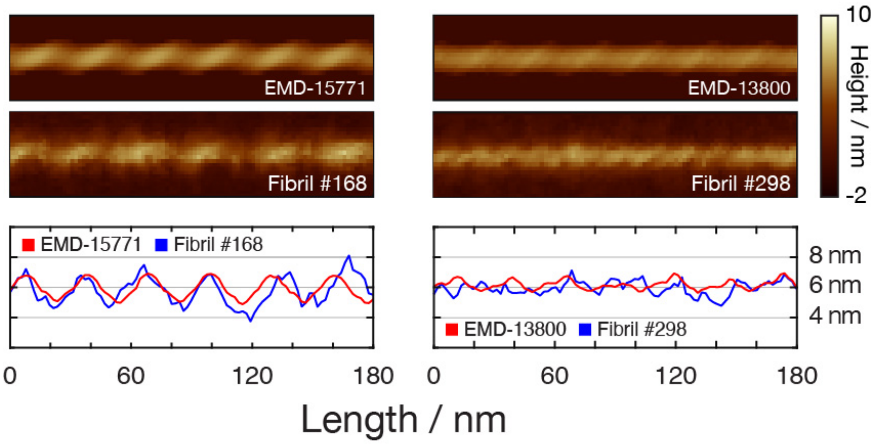

Supplementary Figure S8: Comparison of 1D centre-line sections of simulated AFM images of Type I and Type II Aβ$_{42}$ fibril polymorphs from human patient brain samples with their respective best matched individual fibrils seen in the heterogeneous fibril populations. In the lower AFM height plots, the centre-line height sections of the simulated images (red lines) and their respective best matched filament images (blue lines) obtained from the same images shown in Figure 5 are displayed together to further show the close similarities of the best matched pairs.

Supplementary Table S1: Solution conditions commonly used for Aβ_{42} assembly. Solution conditions used in highly cited primary Aβ_{42} research publications between 2005 and May 2020. The 20 highly cited publications involving Aβ_{42} from each of these years were tabulated and the buffer conditions in which monomer was incubated in which substantial polymerisation could occur are listed.

Title	Publication Year	Corresponding Author(s)	Solution Condition
A hybrid molecule that prohibits amyloid fibrils and alleviates neuronal toxicity induced by β-amyloid (1–42)	2005	Jaehoon Yu	10 mM sodium phosphate at pH 7.4
Certain Inhibitors of Synthetic Amyloid β-Peptide (Aβ) Fibrillogenesis Block Oligomerization of Natural Aβ and Thereby Rescue Long-Term Potentiation	2005	Dennis J. Selkoe	100 mm Tris-HCl, pH 7.4
Protection of rat primary hippocampal cultures from Aβ cytotoxicity by pro-inflammatory molecules is mediated by astrocytes	2005	Rommy von Bernhardi	12 mM Tris buffer, pH 7
Amyloid-β Peptide Inhibits Activation of the Nitric Oxide/cGMP/cAMP-Responsive Element-Binding Protein Pathway during Hippocampal Synaptic Plasticity	2005	Ottavio Arancio	ACSF
Fucoidan inhibits cellular and neurotoxic effects of β-amyloid (Aβ) in rat cholinergic basal forebrain neurons	2005	S. Kar	ACSF/ Neurobasal
Novel Aβ peptide immunogens modulate plaque pathology and inflammation in a murine model of Alzheimer's disease	2005	Charles G. Glabe and Andrea J. Tenner	MilliQ water
γ-Glutamylcysteine ethyl ester protection of proteins from Aβ(1–42)-mediated oxidative stress in neuronal cell culture: A proteomics approach	2005	D. Allan Butterfield	MilliQ water
Modulation of the humoral and cellular immune response in Aβ immunotherapy by the adjuvants monophosphoryl lipid A (MPL), cholera toxin B subunit (CTB) and E. coli enterotoxin LT(R192G)	2005	Cynthia A. Lemere	MilliQ water
β-Amyloid (Aβ) causes detachment of N1E-115 neuroblastoma cells by acting as a scaffold for cell-associated plasminogen activation	2005	Martijn F.B.G. Gebbink	MilliQ water
Neurotoxicity and oxidative stress in D1M-substituted Alzheimer's Aβ(1-42): relevance to N-terminal methionine chemistry in small model peptides	2005	D. Allan Butterfield	MilliQ water
β-Amyloid-Induced Neuronal Apoptosis Involves c-Jun N-Terminal Kinase-Dependent Downregulation of Bcl-w	2005	Christian Pike	MilliQ water
Microglial Phagocytosis Induced by Fibrillar β-Amyloid and IgGs Are Differentially Regulated by Proinflammatory Cytokines	2005	Gary E. Landreth	MilliQ water
Proteomic identification of proteins specifically oxidized by intracerebral injection of amyloid β-peptide (1–42) into rat brain: Implications for Alzheimer's disease	2005	D. Allan Butterfield	MilliQ water
Differential effects of oligomeric and fibrillar amyloid-β1–42 on astrocyte-mediated inflammation	2005	Mary Jo LaDu	Oligomers - Ham's F-12 (phenol red-free) / Fibrils - 10mM HCl
β-Secretase-Cleaved Amyloid Precursor Protein Accumulates at Actin Inclusions Induced in Neurons by Stress or Amyloid β: A Feedforward Mechanism for Alzheimer's Disease	2005	James R. Branburg	Oligomers - Ham's F-12 (phenol red-free) / Fibrils - 10mM HCl

Proteomic identification of proteins oxidized by Aβ(1–42) in synaptosomes: Implications for Alzheimer's disease	2005	D. Allan Butterfield	PBS
Erythropoietin Requires NF-κB and its Nuclear Translocation to Prevent Early and Late Apoptotic Neuronal Injury During β-Amyloid Toxicity	2005	Kenneth Maiese	PBS
Oxidation of cholesterol catalyzed by amyloid β-peptide (Aβ)–Cu complex on lipid membrane	2005	Ryoichi Kuboi	PBS, pH 7.4
Trehalose differentially inhibits aggregation and neurotoxicity of beta-amyloid 40 and 42	2005	Michael R. Sierks	PBS, pH 7.4
A 13 kDa carboxy-terminal fragment of ApoE stabilizes Abeta hexamers	2005	Christian Czech	PBS, pH 7.2
Immunization with amyloid-β using GM-CSF and IL-4 reduces amyloid burden and alters plaque morphology	2006	JoAnne McLaurin	PBS, pH 7.5
Antibodies against β-amyloid reduce aβ oligomers, glycogen synthase kinase-3β activation and τ phosphorylation in vivo and in vitro	2006	Greg M. Cole	10 mM HEPES, pH 7.4
PEGylated phospholipid nanomicelles interact with β-amyloid(1–42) and mitigate its β-sheet formation, aggregation and neurotoxicity in vitro	2006	Hayat Önyüksel	10 mM HEPES
Interaction between Aβ Peptide and α Synuclein: Molecular Mechanisms in Overlapping Pathology of Alzheimer's and Parkinson's in Dementia with Lewy Body Disease	2006	Ratna Mandal	100 mM SDS in 90% MilliQ H_2O and 10% D_2O at pH 7.2
The cAMP-specific phosphodiesterase 4B mediates Aβ-induced microglial activation	2006	Patrick Tremblay	5 mM Tris, pH 7.4
The various aggregation states of β-amyloid 1–42 mediate different effects on oxidative stress, neurodegeneration, and BACE-1 expression	2006	Massimo Tabaton	DMEM
Role of toll-like receptor signalling in Aβ uptake and clearance	2006	Ken - ichiro Fukuchi	Ham's F-12 (phenol red-free)
Autoinsertion of soluble oligomers of Alzheimer's Aβ(1–42) peptide into cholesterol-containing membranes is accompanied by relocation of the sterol towards the bilayer surface	2006	Jeremy P. Bradshaw	Ham's F-12 (phenol red-free)
Autophagy of amyloid beta-protein in differentiated neuroblastoma cells exposed to oxidative stress	2006	Alexei Terman	Ham's F-12 (phenol red-free)
Soluble oligomers of amyloid-β peptide induce neuronal apoptosis by activating a cPLA2-dependent sphingomyelinase-ceramide pathway	2006	Thierry Pillot	Ham's F-12 (phenol red-free)
Alzheimer's amyloid β-peptide (1–42) induces cell death in human neuroblastoma via bax/bcl-2 ratio increase: An intriguing role for methionine 35	2006	F. Misiti	MEM
Low molecular weight thiol amides attenuate MAPK activity and protect primary neurons from Aβ(1–42) toxicity	2006	Daphne Atlas	MilliQ water
β-Amyloid Stimulates Murine Postnatal and Adult Microglia Cultures in a Unique Manner	2006	Colin K. Combs	MilliQ water
The spirostenol (22R, 25R)-20α-spirost-5-en-3β-yl hexanoate blocks mitochondrial uptake of Aβ in neuronal cells and prevents Aβ-induced impairment of mitochondrial function	2006	Vassilios Papaopoulos	MilliQ water, pH 6
Lack of glutathione peroxidase-1 exacerbates Ab-mediated neurotoxicity in cortical neurons	2006	R. C. Iannello	Neurobasal
Apoptosis is secondary to non-apoptotic axonal degeneration in neurons exposed to Aβ in distal axons	2006	Elena I. Posse de Chaves	Oligomers - Ham's F-12 (phenol red-free) / Fibrils - 10mM HCl
Active immunization trial in Aβ42-injected P301L tau transgenic mice	2006	Jürgen Götz	PBS
Immunization with fibrillar Aβ1–42 in young and aged canines: Antibody generation and characteristics, and effects on CSF and brain Aβ	2006	Carl W. Cotman	PBS, pH 7.5
Development of new screening system for Alzheimer disease, in vitro Aβ sink assay, to identify the dissociation of soluble Aβ from fibrils	2006	Ryuichi Morishita	PBS, pH 7.5

Copper-dependent inhibition of cytochrome c oxidase by Aβ1−42 requires reduced methionine at residue 35 of the Aβ peptide	2006	Ian A. Trounce	PBS, pH 8
Aβ peptides can enter the brain through a defective blood–brain barrier and bind selectively to neurons	2007	Robert G. Nagele	0.5 × PBS, 250 mM HEPES buffer, pH 8.5
Single particle detection of Aβ aggregates associated with Alzheimer's disease	2007	Dieter Willbold	140 mM NaCl; 2.7 mM KCl; 10 mM Na_2HPO_4, pH 7.4
Requirement of aggregation propensity of Alzheimer amyloid peptides for neuronal cell surface binding	2007	Avijit Chakrabartty	2 mM borate, 2 mM citrate and 2 mM phosphate, pH 6.5
Lipid peroxidation and 4-hydroxy-2-nonenal formation by copper ion bound to amyloid-β peptide	2007	Masao Nakamura	5 mM Hepes buffer, pH 7.4
Neprilysin protects neurons against Aβ peptide toxicity	2007	Mark S. Kindy	50 mM HEPES buffer, pH 7.2
Dopamine release in prefrontal cortex in response to β-amyloid activation of α7∗ nicotinic receptors	2007	Robert A. Nichols	ACSF
Aβ Upregulates and Colocalizes with LGI3 in Cultured Rat Astrocytes	2007	Yasuhiro Yoshikawa	DMEM
Minocycline does not affect amyloid β phagocytosis by human microglial cells	2007	Robert Veerhuis	DMEM
Natural Oligomers of the Alzheimer Amyloid-β Protein Induce Reversible Synapse Loss by Modulating an NMDA-Type Glutamate Receptor-Dependent Signaling Pathway	2007	Dennis J. Selkoe and Bernado L. Sabatini	Eluted in 50 mm ammonium acetate, pH 8.5
Rapid, concurrent alterations in pre- and postsynaptic structure induced by naturally-secreted amyloid-β protein	2007	Shelley Halpain	Eluted in 50 mm ammonium acetate, pH 8.5
Aβ Oligomer-Induced Aberrations in Synapse Composition, Shape, and Density Provide a Molecular Basis for Loss of Connectivity in Alzheimer's Disease	2007	William L. Klein	Ham's F-12 (phenol red-free)
Aβ Oligomers Induce Neuronal Oxidative Stress through an N-Methyl-D-aspartate Receptor-dependent Mechanism That Is Blocked by the Alzheimer Drug Memantine	2007	Sergio T. Ferreira and William L. Klein	Ham's F-12 (phenol red-free)
Lipoprotein Receptor-Related Protein-1 Mediates Amyloid-β-Mediated Cell Death of Cerebrovascular Cells	2007	Marcel M. Verbeek	MEM
High content screen microscopy analysis of Aβ1–42-induced neurite outgrowth reduction in rat primary cortical neurons: Neuroprotective effects of α7 neuronal nicotinic acetylcholine receptor ligands	2007	Jinhe Li	MEM - F12
Lovastatin protects human neurons against Aβ-induced toxicity and causes activation of β-catenin–TCF/LEF signaling	2007	Francis Amara	MilliQ water
Dopamine and Aβ-Induced Stress Signaling and Decrements in Ca2+ Buffering In Primary Neonatal Hippocampal Cells Are Antagonized by Blueberry Extract	2007	D. R. Fisher	MilliQ water
Soluble amyloid beta1-42 reduces dopamine levels in rat prefrontal cortex: Relationship to nitric oxide	2007	S. Govoni	MilliQ water
Aβ42 neurotoxicity in primary co-cultures: Effect of apoE isoform and Aβ conformation	2007	Mary Jo LaDu	Oligomers - Ham's F-12 (phenol red-free) / Fibrils - 10mM HCl
Oligomeric Amyloid Decreases Basal Levels of Brain-Derived Neurotrophic factor (BDNF) mRNA via Specific Downregulation of BDNF Transcripts IV and V in Differentiated Human Neuroblastoma Cells	2007	Margaret Fahnestock	Oligomers - Ham's F-12 (phenol red-free) / Fibrils - 10mM HCl
Interface peptide of Alzheimer's amyloid beta: Application in purification	2007	Alagiri Srinivasan	PBS
TGF-β1 protects against Aβ-neurotoxicity via the phosphatidylinositol-3-kinase pathway	2008	Agata Copani	0.1 M phosphate-buffered saline
Amyloid-β Peptide (Aβ) Neurotoxicity Is Modulated by the Rate of Peptide Aggregation: Aβ Dimers and Trimers Correlate with Neurotoxicity	2008	Kevin J. Barnham	10 mm Na_2HPO_4 and NaH_2PO_4, pH 7.4
Aβ Mediated Diminution of MTT Reduction—An Artefact of Single Cell Culture?	2008	Marcus Fändrich and Klaus G. Reymann	20 mM NaH2PO4, 140 mM NaCl, 0.2% SDS, pH 7.4
Lipids revert inert Aβ amyloid fibrils to neurotoxic protofibrils that affect learning in mic	2008	Frederic Rousseau	50 mM Tris, pH 7.5

Salvianolic acid B inhibits Aβ fibril formation and disaggregates preformed fibrils and protects against Aβ-induced cytotoxicity	2008	William L. Klein and Min Li	50 mM phosphate buffer pH7.4, and 100 mM NaCl
Aβ inhibits the proteasome and enhances amyloid and tau accumulation	2008	Frank M. LaFerla	50 mM Tris, pH 7.5, 25 mM KCl, 10 mM NaCl, 1 mM MgCl2, 0.03% SDS
Differences between normal and alpha-synuclein overexpressing SH-SY5Y neuroblastoma cells after Aβ(1-42) and NAC treatment	2008	Zsolt L. Datki	DMEM
Aβ Oligomers Induce Neuronal Cell Cycle Events in Alzheimer's Disease	2008	Bruce T. Lamb	Ham's F-12 (phenol red-free)
ApoE Promotes the Proteolytic Degradation of Aβ	2008	Gary E. Landreth	MilliQ water
Blocking Aβ42 Accumulation Delays the Onset and Progression of Tau Pathology via the C Terminus of Heat Shock Protein70-Interacting Protein: A Mechanistic Link between Aβ and Tau Pathology	2008	Frank M. LaFerla	MilliQ water
Aβ(1–42) injection causes memory impairment, lowered cortical and serum BDNF levels, and decreased hippocampal 5-HT2A levels	2008	S. Aznar	MilliQ water
Aggregation of Aβ(1–42)in the presence of short peptides: conformational studies	2008	Botond Penke	MilliQ water (pH 6)
Small Molecule, Non-Peptide p75NTR Ligands Inhibit Aβ-Induced Neurodegeneration and Synaptic Impairment	2008	Frank M. Longo	Oligomers - 0.5 mM NaOH or 0.2% NH_4OH or PBS / Fibrils - 10mM HCl
Oligomer-specific Aβ toxicity in cell models is mediated by selective uptake	2008	Wiep Scheper	Oligomers - DMEM (phenol red - free)/ Fibrils - 10mM HCl
E2-25K/Hip-2 regulates caspase-12 in ER stress–mediated Aβ neurotoxicity	2008	Yong - Keun Jung	PBS
Alzheimer's disease-type neuronal tau hyperphosphorylation induced by Aβ oligomers	2008	Fernanda G De Felice	PBS, pH 7.4
Grape-Derived Polyphenolics Prevent Aβ Oligomerization and Attenuate Cognitive Deterioration in a Mouse Model of Alzheimer's Disease	2008	Giulio M. Pasinetti	PBS, pH 7.4
Anti-oligomeric Aβ Single-chain Variable Domain Antibody Blocks Aβ-induced Toxicity Against Human Neuroblastoma Cells	2008	Michael R. Sierks	PBS, pH 7.4
Aβ-globulomers are formed independently of the fibril pathway	2008	Stefan Barghorn	PBS, pH 7.4
A Two-Year Study with Fibrillar β-Amyloid (Aβ) Immunization in Aged Canines: Effects on Cognitive Function and Brain Aβ	2008	David Cribbs	PBS, pH 7.5
GluN2B subunit-containing NMDA receptor antagonists prevent Aβ-mediated synaptic plasticity disruption in vivo	2009	Michael J. Rowan	0.9% saline
Design and synthesis of newtrehalose-conjugated pentapeptides as inhibitors of Aβ(1–42) fibrillogenesis and toxicity	2009	Enrico Rizzarelli	20 mM PBS or 25 mM NEM buffer, pH 7.4
Comparison of Alzheimer Aβ(1–40) and Aβ(1–42) amyloid fibrils reveals similar protofilament structures	2009	Marcus Fändrich and Nikolaus Grigorieff	50 mM Tris/HCl (pH 7.4)
Aβ-Dependent Inhibition of LTP in Different Intracortical Circuits of the Visual Cortex: The Role of RAGE	2009	Luciano Domenici	ACSF
Preparation and Characterization of a Monoclonal Antibody with High Affinity for Soluble Ab Oligomers	2009	Hong Tao	Ham's F-12 (phenol red-free)
Protection of synapses against Alzheimer's-linked toxins: Insulin signaling prevents the pathogenic binding of Aβ oligomers	2009	William L. Klein	Ham's F-12 (phenol red-free)
The binding of pullulan modified cholesteryl nanogels to Aβ oligomers and their suppression of cytotoxicity	2009	Dusica Maysinger	Ham's F-12 (phenol red-free)
CD14 and Toll-Like Receptors 2 and 4 Are Required for Fibrillar Aβ-Stimulated Microglial Activation	2009	Gary E. Landreth	MilliQ water
Autophagy protects neuron from Aβ-induced cytotoxicity	2009	Wen-Mei Fu	MilliQ water
TrkA pathway activation induced by amyloid-beta (Abeta)	2009	Massimo Masserini	Neurobasal, pH 7.4

Pin1 affects Tau phosphorylation in response to Aβ oligomers	2009	Massimo Masserini	Neurobasal, pH 7.4
Enlargement of Aβ aggregates through chemokine-dependent microglial clustering	2009	Huey - Jen Tsay	Oligomers - Ham's F-12 (phenol red-free) / Fibrils - 10mM HCl and 150mM NaCl
PP1 inhibition by Aβ peptide as a potential pathological mechanism in Alzheimer's disease	2009	Edgar F. da Cruz e Silva	Oligomers - possibly cell culture medium / Fibrils - 10mM HCl
Aβ peptide conformation determines uptake and interleukin-1α expression by primary microglial cells	2009	Greer M. Murphy Jr.	Oligomers and Fibrils - PBS / Protofibrils - Ham's F12 (all pH 7.4)
Human apolipoprotein A–I binds amyloid-β and prevents Aβ-induced neurotoxicity	2009	Sergio T. Ferreira	PBS
Single-domain antibodies recognize selectively small oligomeric forms of amyloid β, prevent Aβ-induced neurotoxicity and inhibit fibril formation	2009	François Rougeon	PBS
Diazoxide Reverses the Enhanced Expression of KATP Subunits in Cholinergic Neurons Caused by Exposure to Aβ1-42	2009	Kejing Liu	PBS
A Role for Synaptic Zinc in Activity-Dependent Aβ Oligomer Formation and Accumulation at Excitatory Synapses	2009	Jorge Busciglio	PBS, pH 7.4
Characterisation of two antibodies to oligomeric Aβ and their use in ELISAs on human brain tissue homogenates	2009	Seth Love	PBS; 0.15 M sodium chloride, 1.9 mM sodium dihydrogen orthophosphate 1-hydrate, 7.5 mM di-sodium hydrogen orthophosphate 12-hydrate, pH 7.1) or Dulbecco's Modified Eagle Medium
The PI3K-Akt-mTOR pathway regulates Aβ oligomer induced neuronal cell cycle events	2009	Bruce T. Lamb	Sodium phosphate buffer at pH 7.4
Hydralazine modifies Aβ fibril formation and prevents modification by lipids in vitro	2010	Ian V. J. Murray	100mM PBS, pH 7.4
Aβ20–29 peptide blocking apoE/Aβ interaction reduces full-length Aβ42/40 fibril formation and cytotoxicity in vitro	2010	Zhuyi Li	100mM Tris, pH 7.4
TGF-β1 blockade of microglial chemotaxis toward Aβ aggregates involves SMAD signaling and down-regulation of CCL5	2010	Huey - Jen Tsay	10mM HCl and 150mM NaCl
Stability of Aβ (1-42) peptide fibrils as consequence of environmental modifications	2010	Francesco Mantegazza	10mM HCl and switched to various buffers at pH 7.4 or 10 mM Tris HCl, 150 mM NaCl, pH 7.4
Retardation of Aβ Fibril Formation by Phospholipid Vesicles Depends on Membrane Phase Behavior	2010	Sara Linse	20 mM Tris/HCl, pH 7.4, 0.2 mM EDTA, 0.02% NaN_3
Surface Plasmon Resonance Spectroscopy in Determination of the Interactions Between Amyloid β Proteins (Aβ) and Lipid Membranes	2010	Marie - Isabel Aguilar	20 mM sodium phosphate (pH 6.8)
The Native Copper- and Zinc- Binding Protein Metallothionein Blocks Copper-Mediated Aβ Aggregation and Toxicity in Rat Cortical Neurons	2010	Adrian K. West	20mM Tris-HCl, 100mM NaCl, pH 7.4
Sensitive detection of Aβ protofibrils by proximity ligation - relevance for Alzheimer's disease	2010	Ulf Landegren	50 mM phosphate buffer and 100 mM NaCl, pH 7.4
Preparation and characterization of toxic Aβ aggregates for structural and functional studies in Alzheimer's disease research	2010	Hilal A. Lashuel	500 mM glycine–NaOH, pH 8.5
Amyloid beta from axons and dendrites reduces local spine number and plasticity	2010	Roberto Malinow	ACSF
Aβ Oligomers Cause Localized Ca2+ Elevation, Missorting of Endogenous Tau into Dendrites, Tau Phosphorylation, and Destruction of Microtubules and Spines	2010	Eckhard and Eva Mandelkow	Ham's F-12 (phenol red-free)
Soluble oligomeric forms of beta-amyloid (Aβ) peptide stimulate Aβ production via astrogliosis in the rat brain	2010	A. Gonzalo - Ruiz	Ham's F-12 (phenol red-free)
Activation of PERK Signaling Attenuates Aβ-Mediated ER Stress	2010	Sung Su Kim	MilliQ water
Human intravenous immunoglobulin provides protection against Aβ toxicity by multiple mechanisms in a mouse model of Alzheimer's disease	2010	Milla Koistinaho	MilliQ water

Conformation dependent monoclonal antibodies distinguish different replicating strains or conformers of prefibrillar Aβ oligomers	2010	Charles Glabe	MilliQ water/ HEPES buffered saline, pH 7.4 (HBS) / phosphate buffered saline pH 7.4 (PBS) all supplemented with 0.02% NaN_3
Synthetic amyloid-β oligomers impair long-term memory independently of cellular prion protein	2010	Gianluigi Forloni	Oligomers - 50 mM phosphate buffer, 150 mM NaCl, pH 7.4 / Fibrils - 10mM HCl
Selenium attenuates Aβ production and Aβ-induced neuronal death	2010	Dong - Gyu Jo	PBS
Stabilization of neurotoxic Alzheimer amyloid-β oligomers by protein engineering	2010	Christopher Dobson and Torleif Hard	PBS, pH 7.2
Linear and conformation specific antibodies in aged beagles after prolonged vaccination with aggregated Abeta	2010	Elizabeth Head	PBS, pH 7.5
The presence of sodium dodecyl sulphate-stable Aβ dimers is strongly associated with Alzheimer-type dementia	2010	Dominic Walsh	Tris-buffered saline (TBS), containing 5 mM ethylenediaminetetraacetic acid (EDTA), 5 mM ethylene glycol tetraacetic acid (EGTA), 10 mg/ml leupeptin, 1 mg/ml aprotinin, 1 mg/ml pepstatin A, 1 mM Pefabloc, 2 mM 1,10-phenanthroline
The Osaka FAD Mutation E22Δ Leads to the Formation of a Previously Unknown Type of Amyloid β Fibrils and Modulates Aβ Neurotoxicity	2011	Rudi Glockshuber	10 mM H_3PO_4-NaOH, pH 7.4, 100 mM NaCl
Critical Role of Astroglial Apolipoprotein E and Liver X Receptor-α Expression for Microglial Aβ Phagocytosis	2011	Michael T. Heneka	50 mM Tris-HCl, pH 7
Resveratrol Acts Not through Anti-Aggregative Pathways but Mainly via Its Scavenging Properties against Aβ and Aβ-Metal Complexes Toxicity	2011	Paolo Zatta	Dialyzed against various metal solutions 10 mM ($[CH3CH(OH)COO]_3Al$, $FeCl_3$, $CuCl_2$, $ZnCl_2$) and then bidialyzed x3 in MilliQ water, pH 7. *In vitro* preps were diluted into 0.1 M Tris/HCl pH 7.4 buffer plus 0.15 M NaCl
Abeta Peptide Toxicity is Reduced After Treatments Decreasing Phosphatidylethanolamine Content in Differentiated Neuroblastoma Cells	2011	Massimo Masserini	DMEM/ PBS
Mechanism mediating oligomeric Aβ clearance by naïve primary microglia	2011	Huey - Jen Tsay	Ham's F-12 (phenol red-free)
The Second-Generation Active Aβ Immunotherapy CAD106 Reduces Amyloid Accumulation in APP Transgenic Mice While Minimizing Potential Side Effects	2011	Matthias Staufenbiel	Ham's F-12 (phenol red-free)
Cognitive effects of cell-derived and synthetically derived Aβ oligomers	2011	James P. Cleary	Ham's F-12 (phenol red-free)
Aβ Inhibition of Ionic Conductance in Mouse Basal Forebrain Neurons Is Dependent upon the Cellular Prion Protein PrPC	2011	Jack H. Jhamandas	Ham's F-12 (phenol red-free)
Membrane cholesterol enrichment prevents Aβ-induced oxidative stress in Alzheimer's fibroblasts	2011	Cristina Cecchi	Ham's F-12 (phenol red-free)
Specificity and sensitivity of the Abeta oligomer ELISA	2011	David A. Loeffler	LMW - 100mM Tris, pH 8.8 Oligomers - PBS; 0.01 M, pH 7.4, with 0.02% azide
Protection against Aβ-mediated rapid disruption of synaptic plasticity and memory by memantine	2011	Michael J. Rowan	MilliQ water
p75NTR Regulates Aβ Deposition by Increasing Aβ Production But Inhibiting Aβ Aggregation with Its Extracellular Domain	2011	Xin - Fu Zhou	Oligomers - DMEM Fibrils - DMEM with 10mM HCl
WNT5A Signaling Contributes to Aβ-Induced Neuroinflammation and Neurotoxicity	2011	Shao - Jun Tang	Oligomers - DMEM/F-12 Fibrils - 10mM HCl
The contribution of activated astrocytes to Aβ production: Implications for Alzheimer's disease pathogenesis	2011	Robert Vassar	Oligomers - Ham's F-12 (phenol red-free), Fibrils -10mM HCl

Downregulation of CREB expression in Alzheimer's brain and in Aβ-treated rat hippocampal neurons	2011	Christopher B. Eckman	Oligomers - Ham's F-12 (phenol red-free), Fibrils -10mM HCl
Functional Links Between Aβ Toxicity, Endocytic Trafficking, and Alzheimer's Disease Risk Factors in Yeast	2011	Susan Lindquist	Oligomers - MilliQ water Fibrils - 10mM HCL 6% DMSO
Lipid matrix plays a role in Abeta fibril kinetics and morphology	2011	Frances Separovic	PBS
Sinomenine inhibits microglial activation by Aβ and confers neuroprotection	2011	Shiv Kumar Sharma	PBS
Soluble Aβ Seeds Are Potent Inducers of Cerebral β-Amyloid Deposition	2011	Mathias Jucker	PBS, pH 7
Silibinin: A novel inhibitor of Aβ aggregation	2011	Junzeng Zhang	PBS, pH 7.4
ERK and p38 inhibitors attenuate memory deficits and increase CREB phosphorylation and PGC-1α levels in Aβ-injected rats	2012	Fariba Khodagholi	0.1 M PBS
Aβ neurotoxicity depends on interactions between copper ions, prion protein, and N-methyl-d-aspartate receptors	2012	Gerald W. Zamponi	0.9 % saline, pH 7.4
The phosphodiesterase-4 inhibitor rolipram reverses Aβ-induced cognitive impairment and neuroinflammatory and apoptotic responses in rats	2012	Han - Ting Zhang	0.9% saline
Amyloid-Beta (Aβ) D7H Mutation Increases Oligomeric Aβ42 and Alters Properties of Aβ-Zinc/Copper Assemblies	2012	Irene H. Cheng	10 mM Tris-HCl, pH 7.4
The binding of resveratrol to monomer and fibril amyloid beta	2012	Jiang - Ning Zhou	50 mM phosphate buffer, pH 7.5, and 100 mM NaCl
Specific Binding of Alzheimer's Aβ Peptide Fibrils to Single-Walled Carbon Nanotubes	2012	Saeyoung Nate Ahn	50mM MOPS, pH 7.4
Aβ delays fibrin clot lysis by altering fibrin structure and attenuating plasminogen binding to fibrin	2012	Sidney Strickland	50mM Tris pH 7.4, 0.1% NH_4OH
p75NTR is mainly responsible for Aβ toxicity but not for its internalization: a primary study	2012	Xin - Fu Zhou	DMEM
Synapse-Binding Subpopulations of Aβ Oligomers Sensitive to Peptide Assembly Blockers and scFv Antibodies	2012	William L. Klein	Ham's F-12 (phenol red-free)
Aβ Oligomers-Induced Toxicity is Attenuated in Cells Cultured with NbActiv4™ Medium	2012	William L. Klein	Ham's F-12 (phenol red-free)
Redox-active Cu(II)–Aβ causes substantial changes in axonal integrity in cultured cortical neurons in an oxidative-stress dependent manner	2012	Roger S. Chung	MilliQ water
Aβ-induced formation of autophagosomes is mediated by RAGE-CaMKKβ-AMPK signaling	2012	Inhee Mook-Jung	Opti-MEM
An Effector-Reduced Anti-β-Amyloid (Aβ) Antibody with Unique Aβ Binding Properties Promotes Neuroprotection and Glial Engulfment of Aβ	2012	Andreas Muhs and Ryan J. Watts	PBS
NMDA receptors and BAX are essential for Aβ impairment of LTP	2012	Morgan Sheng	PBS
A critical role for the PAR-1/MARK-tau axis in mediating the toxic effects of Aβ on synapses and dendritic spines	2012	Bingwei Li	PBS
Neuropathogenic role of adenylate kinase-1 in Aβ-mediated tau phosphorylation via AMPK and GSK3β	2012	Yong - Keun Jung	PBS
The Microtubule-Associated Protein 1A (MAP1A) is an Early Molecular Target of Soluble Aβ-Peptide	2012	A. B. Klein	PBS containing 0.1% NH_4OH
A Chemical Analog of Curcumin as an Improved Inhibitor of Amyloid Abeta Oligomerization	2012	David L. Vander Jagt	PBS, pH 7.2
An anti-diabetes agent protects the mouse brain from defective insulin signaling caused by Alzheimer's disease–associated Aβ oligomers	2012	Fernanda G De Felice	PBS, pH 7.4
Interaction between NH2-tau fragment and Aβ in Alzheimer's disease mitochondria contributes to the synaptic deterioration	2012	Pietro Calissano	PBS, pH 7.4
Inhibition of Phosphorylation of JNK Suppresses Aβ-Induced ER Stress and Upregulates Prosurvival Mitochondrial Proteins in Rat Hippocampus	2013	Fatemeh Shaerzadeh	0.1 M PBS

Aβ Association Inhibition by Transferrin	2013	Giuseppe Melacini	15 mM potassium phosphate buffer at pH 7.4, with 10 % D_2O and 0.02 % NaN_3
Proteolytically Inactive Insulin-Degrading Enzyme Inhibits Amyloid Formation Yielding Non-Neurotoxic Aβ Peptide Aggregates	2013	Eduardo M. Castaño	50 mM NaCl, 20 mM Tris-HCl, pH 7.4
Ammonium hydroxide treatment of Aβ produces an aggregate free solution suitable for biophysical and cell culture characterization	2013	Blaine R. Roberts	60mM NaOH - further diluted into: PBS, pH 7.4 - ThT / 50mM Tris, 10mM EDTA, pH 8 - DLS
Intracellular accumulation of aggregated pyroglutamate amyloid beta: convergence of aging and Aβ pathology at the lysosome	2013	Wiep Scheper	DMEM (phenol red - free)
Exosomes neutralize synaptic-plasticity-disrupting activity of Aβ assemblies in vivo	2013	Joung - Hun Kim	Ham's F-12 (phenol red-free)
Disruption of neocortical histone H3 homeostasis by soluble Aβ: implications for Alzheimer's disease	2013	Caterina M. Hernandez	Ham's F-12 (phenol red-free)
The CAMKK2-AMPK Kinase Pathway Mediates the Synaptotoxic Effects of Aβ Oligomers through Tau Phosphorylation	2013	Franck Polleux	Ham's F-12 (phenol red-free)
Blocking the Interaction between Apolipoprotein E and Aβ Reduces Intraneuronal Accumulation of Aβ and Inhibits Synaptic Degeneration	2013	Martin J. Sadowski	Ham's F-12 (phenol red-free)
Protection against the synaptic targeting and toxicity of Alzheimer's-associated Aβ oligomers by insulin mimetic chiro-inositols	2013	William L. Klein	Ham's F-12 (phenol red-free)
Leptin and ghrelin prevent hippocampal dysfunction induced by Aβ oligomers	2013	C.M.F. Pereira	Ham's F-12 (phenol red-free)
Aβ leads to Ca2+ signaling alterations and transcriptional changes in glial cells	2013	Dmitry Lim	Ham's F-12 (phenol red-free)
Canonical Wnt signaling protects hippocampal neurons from Aβ oligomers: role of non-canonical Wnt-5a/Ca2+ in mitochondrial dynamics	2013	Nibaldo C Inestroasa	MilliQ water
Oligomeric Aβ-Induced Microglial Activation is Possibly Mediated by NADPH Oxidase	2013	Chun Fu Wu	MilliQ water
The cellular prion protein traps Alzheimer's Aβ in an oligomeric form and disassembles amyloid fibers	2013	John H. Viles	Monomer - Water, pH 10.5 - maintained using NaOH<br>Fibril elongation - 160mM NaCl, 30mM HEPES, pH 7.4
Memantine Rescues Transient Cognitive Impairment Caused by High-Molecular-Weight Aβ Oligomers But Not the Persistent Impairment Induced by Low-Molecular-Weight Oligomers	2013	Sergio T. Ferreira	PBS
Immobilized amyloid Aβ peptides support platelet adhesion and activation	2013	Mauro Torti	PBS
Characterization of a Single-Chain Variable Fragment Recognizing a Linear Epitope of Aβ: A Biotechnical Tool for Studies on Alzheimer's Disease?	2013	Dieter Willbold	PBS
Peripherally Applied Synthetic Peptide isoAsp7-Aβ(1-42) Triggers Cerebral β-Amyloidosis	2013	A.A. Makarov	PBS, pH 7, further diluted in surgical saline
Curcumin-conjugated nanoliposomes with high affinity for Aβ deposits: Possible applications to Alzheimer disease	2013	Charles Duyckaerts	PBS, with or without 10% Curcumin-conjugated nanoliposomes (CnLs), (20 µg/ml final concentration)
Schisantherin A recovers Aβ-induced neurodegeneration with cognitive decline in mice	2014	Ying Jia	0.9% saline
Aβ induces its own prion protein N-terminal fragment (PrPN1)–mediated neutralization in amorphous aggregates	2014	Xavier Roucou	10 mM Na_2HPO_4, pH 7.4
Distinct synthetic Aβ prion strains producing different amyloid deposits in bigenic mice	2014	Stanley B. Prusiner	10 mM sodium phosphate (NaP) buffer at neutral pH 7.4 with or without 3.47 mM [0.1% (wt/vol)] SDS (Comparison)
Immobilization of Homogeneous Monomeric, Oligomeric and Fibrillar Aβ Species for Reliable SPR Measurements	2014	Dieter Willbold	10 mM sodium phosphate buffer pH 7.4

Aβ-AGE aggravates cognitive deficit in rats via RAGE pathway	2014	S. Zhang	10mM PBS, pH 7.2
Self-Propagative Replication of Aβ Oligomers Suggests Potential Transmissibility in Alzheimer Disease	2014	Vijayaraghavan Rangachari	20 mM Tris-HCl, pH 8.0
Soluble Aβ Oligomers Are Rapidly Sequestered from Brain ISF In Vivo and Bind GM1 Ganglioside on Cellular	2014	Dennis J. Selkoe	50 mM ammonium acetate, pH 8.5
Interaction between Prion Protein and Aβ Amyloid Fibrils Revisited	2014	Witold K. Surewicz	7.5mM PBS, pH 7.4
Aβ promotes VDAC1 channel dephosphorylation in neuronal lipid rafts. Relevance to the mechanisms of neurotoxicity in Alzheimer's disease	2014	R. Marin	DMEM
A Sensitive Aβ Oligomer Assay Discriminates Alzheimer's and Aged Control Cerebrospinal Fluid	2014	Alexander McCampbell	Ham's F-12 (phenol red-free)
Enhancing Astrocytic Lysosome Biogenesis Facilitates Aβ Clearance and Attenuates Amyloid Plaque Pathogenesis	2014	Jin-Moo Lee	Ham's F-12 (phenol red-free)
Elucidating Molecular Mass and Shape of a Neurotoxic Aβ Oligomer	2014	William L. Klein	Ham's F-12 (phenol red-free)
Neurotrophin receptor p75 mediates the uptake of the amyloid beta (Aβ) peptide, guiding it to lysosomes for degradation in basal forebrain cholinergic neurons	2014	J. Oliver Dolly	Ham's F-12 (phenol red-free)
PKCε Promotes HuD-Mediated Neprilysin mRNA Stability and Enhances Neprilysin-Induced Aβ Degradation in Brain Neurons	2014	Daniel Alkon	MilliQ water
Interactions between Aβ oligomers and presynaptic cholinergic signaling: Age-dependent effects on attentional capacities	2014	Britney Yegla	MilliQ water followed by 1 mM $NaHCO_3$, pH 10 after aggregation step
Cyclophilin D deficiency rescues Aβ-impaired PKA/CREB signaling and alleviates synaptic degeneration	2014	Shirley ShiDu Yan	PBS
Intracellular Accumulation of Amyloid-β (Aβ) Protein Plays a Major Role in Aβ-Induced Alterations of Glutamatergic Synaptic Transmission and Plasticity	2014	Claudio Grassi	PBS
Aβ and tau toxicities in Alzheimer's are linked via oxidative stress-induced p38 activation: Protective role of vitamin E	2014	J.Viña	PBS
Clioquinol promotes the degradation of metal-dependent amyloid-β (Aβ) oligomers to restore endocytosis and ameliorate Aβ toxicity	2014	Susan Lindquist	PBS, pH 7.2
Liposomes bi-functionalized with phosphatidic acid and an ApoE-derived peptide affect Aβ aggregation features and cross the blood–brain-barrier: Implications for therapy of Alzheimer disease	2014	Francesca Re	PBS, pH 2
Single Chain Variable Fragment Against Aβ Expressed in Baculovirus Inhibits Abeta Fibril Elongation and Promotes its Disaggregation	2015	Tao Hung	0.1 M boric acid, 2.5 mM NaCl, 2.5 mM sodium borate, pH 8.5
LC3 overexpression reduces Aβ neurotoxicity through increasing α7nAchR expression and autophagic activity in neurons and mice	2015	Wen-Mei Fu	10 mM sodium phosphate buffer
QIAD assay for quantitating a compound's efficacy in elimination of toxic Aβ oligomers	2015	Dieter Willbold	10 mM sodium phosphate buffer, pH 7.4
SDS-PAGE analysis of Aβ oligomers is disserving research into Alzheimer´s disease: appealing for ESI-IM-MS	2015	Natàlia Carulla	10 mM sodium phosphate, pH 7.4
MicroRNAs 99b-5p/100-5p Regulated by Endoplasmic Reticulum Stress are Involved in Abeta-Induced Pathologies	2015	Jun Wan	20mM PBS
Donepezil attenuates Aβ-associated mitochondrial dysfunction and reduces mitochondrial Aβ accumulation in vivo and in vitro	2015	Hai Yang Zhang	250 mM surcrose, 10 mM Tris, 2.5 mM KH2PO4, PH 7.4
Peptide dimer structure in an Aβ(1–42) fibril visualized with cryo-EM	2015	Marcus Fändrich and Nikolaus Grigorieff	50 mM Tris·HCl, pH 7.4
Steady-state and time-resolved Thioflavin-T fluorescence can report on morphological differences in amyloid fibrils formed by Aβ(1-40) and Aβ(1-42)	2015	Elin K. Esbjörner	50 mM sodium phosphate buffer, pH 7.4

Aβ and NMDAR activation cause mitochondrial dysfunction involving ER calcium release	2015	A. Cristina Rego	Ham’s F-12 (phenol red-free)
Aβ-dependent reduction of NCAM2-mediated synaptic adhesion contributes to synapse loss in Alzheimer’s disease	2015	Lars M. Ittner, Vladimir Sytnyk	Ham’s F-12 (phenol red-free)
Dendritic and axonal mechanisms of Ca2+ elevation impair BDNF transport in Aβ oligomer–treated hippocampal neurons	2015	Michael A. Silverman	Ham’s F-12 (phenol red-free)
Cu2+ accentuates distinct misfolding of Aβ(1–40) and Aβ(1–42) peptides, and potentiates membrane disruption	2015	John H. Viles	Water at pH 10.5, using NaOH
Resveratrol inhibits oligomeric Aβ-induced microglial activation via NADPH oxidase	2015	Wei-Qi Li	MilliQ water
Involvement of Intracellular and Mitochondrial Aβ in the Ameliorative Effects of Huperzine A against Oligomeric Aβ42-Induced Injury in Primary Rat Neurons	2015	Hai Yang Zhang	Neurobasal
Minocycline attenuates Aβ oligomers-induced pro-inflammatory phenotype in primary microglia while enhancing Aβ fibrils phagocytosis	2015	Nabila Hamdi	Oligomers - Ham's F12, Fibrils - PBS
Aβ-induced degradation of BMAL1 and CBP leads to circadian rhythm disruption in Alzheimer’s disease	2015	Inhee Mook-Jung	Opti-MEM
Alzheimer-associated Aβ oligomers impact the central nervous system to induce peripheral metabolic deregulation	2015	Fernanda G De Felice	PBS
Stabilization of Nontoxic Aβ-Oligomers: Insights into the Mechanism of Action of Hydroxyquinolines in Alzheimer's Disease	2015	Colin L. Masters	PBS
ApoE4 and Aβ Oligomers Reduce BDNF Expression via HDAC Nuclear Translocation	2015	Daniel Alkon	PBS without Ca^{2+} or Mg^{2+}
Lycopene abrogates Aβ(1–42)-mediated neuroinflammatory cascade in an experimental model of Alzheimer’s disease	2015	Kanwaljit Chopra	PBS, pH 7.4
Rapid α-oligomer formation mediated by the Aβ C terminus initiates an amyloid assembly pathway	2016	Ronald Wetzel	1 x PBS, pH 7.4
Heterotypic seeding of Tau fibrillization by pre-aggregated Abeta provides potent seeds for prion-like seeding and propagation of Tau-pathology in vivo	2016	Diederik Moechars and Ilse Dewachter	10 mM HCl solution
The new β amyloid-derived peptide Aβ1–6A2V-TAT(D) prevents Aβ oligomer formation and protects transgenic C. elegans from Aβ toxicity	2016	Mario Salmona	10 mM phosphate buffer containing 150 mM NaCl, pH 7.4
Astrocytes from old Alzheimer's disease mice are impaired in Aβ uptake and in neuroprotection	2016	Dan Frenkel	10 mM HEPES, 300 mM NaCl, in DDW, pH = 7.4
Atomic-resolution structure of a disease-relevant Aβ(1–42) amyloid fibril	2016	Beat H. Meier and Roland Riek	100 mM phosphate at pH of 7.4, 0/100 mM NaCl, 0/10/100 µM ZnCl / 30 µM heparin. Either zinc or heperin not both.
Structure of Crenezumab Complex with Aβ Shows Loss of β-Hairpin	2016	Weiru Wang	100 mM sodium phosphate buffer, pH 7.4
Mechanisms of tau and Aβ-induced excitotoxicity	2016	Gail V.W. Johnson	135 mM NaCl, 5.4 mM KCl, 0.33 mM NaH_2PO_4, 0.4 mM KH_2PO_4, 20 mM HEPES, 0.8 mM $MgCl_2$, 1.2 mM $CaCl_2$, 10 mM glucose, and pH 7.4
Quantitative analysis of intrinsic and extrinsic factors in the aggregation mechanism of Alzheimer-associated Aβ-peptide	2016	Sara Linse	20 mM sodium phosphate buffer, 200 µM EDTA, 0.02% NaN_3 , pH 8
Presynaptic dystrophic neurites surrounding amyloid plaques are sites of microtubule disruption, BACE1 elevation, and increased Aβ generation in Alzheimer’s disease	2016	Robert Vassar	4 mM HEPES, pH 8
Soluble Conformers of Aβ and Tau Alter Selective Proteins Governing Axonal Transport	2016	Sylvain E. Lesné	50 mm Tris-HCl, 150 mm NaCl, 0.01% Triton X-100, pH 7.4
Soluble prion protein and its N-terminal fragment prevent impairment of synaptic plasticity by Aβ	2016	Witold K. Surewicz	50 mM sodium phosphate buffer, pH 7.4

oligomers: Implications for novel therapeutic strategy in Alzheimer's disease			
Aβ-Induced Synaptic Alterations Require the E3 Ubiquitin Ligase Nedd4-1	2016	Gentry N. Patrick	MEM
The Tyr216 phosphorylated form of GSK3β contributes to tau phosphorylation at PHF-1 epitope in response to Aβ in the nucleus of SH-SY5Y cells	2016	Sabrina Ingrand	MiliQ Water
Dopamine agonists rescue Aβ–induced LTP impairment by Src-family tyrosine kinases	2016	Klaus G. Reymann	Oligomers - Ham's F12, Fibrils - Neurobasal
Identification of a Small Molecule Cyclophilin D Inhibitor for Rescuing Aβ-Mediated Mitochondrial Dysfunction	2016	Shirley ShiDu Yan	PBS
Antibody modified-silver nanoparticles for colorimetric immuno sensing of Aβ(1–40/1–42) based on the interaction between β-amyloid and Cu2+	2016	Xiurong Yang	PBS (50 mM NaH2PO4, 100 mM NaCl, pH 7.6)
Conversion of Synthetic Aβ to In Vivo Active Seeds and Amyloid Plaque Formation in a Hippocampal Slice Culture Model	2016	Mathias Jucker	PBS (added to cells so presumably pH 7.4)
Deacetylation of TFEB promotes fibrillar Aβ degradation by upregulating lysosomal biogenesis in microglia	2016	Jianguo Ji	PBS (added to cells so presumably pH 7.4)
RXR controlled regulatory networks identified in mouse brain counteract deleterious effects of Aβ oligomers	2016	Radosveta Koldamova	serum-free medium
Characterization of insulin-degrading enzyme-mediated cleavage of Aβ in distinct aggregation states	2016	Kerensa Broerson	ThT/TEM - PBS, pH 7.4 NMR - 50 mM phosphate buffer pH 6.8 containing 50 mM NaCl
Novel Curcumin loaded nanoparticles engineered for Blood-Brain Barrier crossing and able to disrupt Abeta aggregates	2017	Andreas M. Grabrucker	ThT - 'Assay buffer', Cell culture - NB+++
Astrocytic LRP1 Mediates Brain Aβ Clearance and Impacts Amyloid Deposition	2017	Guojun Bu	DMEM with 10% FBS
Aβ accumulation causes MVB enlargement and is modelled by dominant negative VPS4A	2017	Gunnar K Gouras	
High-resolution bioelectrical imaging of Aβ-induced network dysfunction on CMOS-MEAs for neurotoxicity and rescue studies	2017	Luca Berdondini	10 mM phosphate buffer, pH 7.4
The Aβ oligomer eliminating D-enantiomeric peptide RD2 improves cognition without changing plaque pathology	2017	Dieter Willbold	10 mM sodium phosphate buffer, pH 7.4.
Human Brain-Derived Aβ Oligomers Bind to Synapses and Disrupt Synaptic Activity in a Manner That Requires APP	2017	Tara Spires - Jones and Dominic Walsh	124 mM NaCl, 2.8 mM KCl, 1.25 mM NaH_2PO_4, and 26 mM $NaHCO_3$, pH 7.4.
A vaccine with Aβ oligomer-specific mimotope attenuates cognitive deficits and brain pathologies in transgenic mice with Alzheimer's disease	2017	Rui-tian Liu	20mM PBS, pH 7.4
Nec-1 alleviates cognitive impairment with reduction of Aβ and tau abnormalities in APP/PS1 mice	2017	YoungSoo Kim	DMEM with 0.5%FBS
Increased susceptibility to Aβ toxicity in neuronal cultures derived from familial Alzheimer's disease (PSEN1-A246E) induced pluripotent stem cells	2017	Claudio Soto	DMEM/F12 without serum
Norovirus P particle-based active Aβ immunotherapy elicits sufficient immunogenicity and improves cognitive capacity in a mouse model of Alzheimer's disease	2017	Wei Kong	Ham's F-12, ThT - 10 mM PB, 500 mM NaCl, pH 7.0
Inhibition of Drp1 Ameliorates Synaptic Depression, Aβ Deposition, and Cognitive Impairment in an Alzheimer's Disease Model	2017	Dong-Gyu Jo	Ham's F-12 (phenol red-free)
Role of membrane GM1 on early neuronal membrane actions of Aβ during onset of Alzheimer's disease	2017	Luis Aguayo	MiliQ water
High-density lipoproteins suppress Aβ-induced PBMC adhesion to human endothelial cells in bioengineered vessels and in monoculture	2017	Cheryl L. Wellington	Monomer - DMEM Oligomer - Ham's F-12 (phenol red-free) Fibrils - 0.1µM HCl.
Strain-specific Fibril Propagation by an Aβ Dodecamer	2017	Vijayaraghavan Rangachari	Monomer - implied PBS LFAO - 50 mM NaCl and 5 mM C12:0 fatty

			acid, Fibrils - 150 mM NaCl with 0.01% NaN_3
Astrocyte Transforming Growth Factor Beta 1 Protects Synapses against Aβ Oligomers in Alzheimer's Disease Model	2017	Flávia Carvalho Alcantara Gomes	PBS
Aβ seeding potency peaks in the early stages of cerebral β-amyloidosis	2017	Mathias Jucker	PBS (10%, w/v)
Colorimetric sandwich immunosensor for Aβ(1-42) based on dual antibody-modified gold nanoparticles	2017	Xiurong Yang	PBS (50 mM NaH_2PO_4, 100 mM NaCl), pH 7.4
Involvement of GluN2B subunit containing N-methyl-d-aspartate (NMDA) receptors in mediating the acute and chronic synaptotoxic effects of oligomeric amyloid-beta (Aβ) in murine models of Alzheimer's disease (AD)	2017	Chris Parsons	Ringer's solution: 125 mM NaCl, 2.5 mM KCl, 25 mM $NaHCO_3$, 2 mM $CaCl_2$, 1 mM $MgCl_2$, 25 mM d-glucose, and 1.25 mM NaH2PO4, bubbled with a 95% O_2, 5% CO_2 mixture, pH of 7.3
Anthocyanin-Loaded PEG-Gold Nanoparticles Enhanced the Neuroprotection of Anthocyanins in an Aβ1–42 Mouse Model of Alzheimer's Disease	2017	Myeong Ok Kim	Saline
A Novel, Multi-Target Natural Drug Candidate, Matrine, Improves Cognitive Deficits in Alzheimer's Disease Transgenic Mice by Inhibiting Aβ Aggregation and Blocking the RAGE/Aβ Axis	2017	Bin Zhao	ThT - 50-mM phosphate buffer (pH 6.0). Other experiments unclear
Amyloid β-Derived Diffusible Ligands (ADDLs) Induce Abnormal Autophagy Associated with Aβ Aggregation Degree	2018	Tao Hung	DMEM/F12 medium without phenol red
Inhibition of Phosphodiesterase-4 Reverses Aβ-Induced Memory Impairment by Regulation of HPA Axis Related cAMP Signaling	2018	Jiangchun Pan	0.9% saline
The Aβ protofibril selective antibody mAb158 prevents accumulation of Aβ in astrocytes and rescues neurons from Aβ-induced cell death	2018	Anna Erlandsson	10X PBS
Diffusible, highly bioactive oligomers represent a critical minority of soluble Aβ in Alzheimer's disease brain	2018	Dominic Walsh	124 mM NaCl, 2.8 mM KCl, 1.25 mM NaH_2PO_4, 26 mM $NaHCO_3$, pH 7.4
Aβ truncated species: Implications for brain clearance mechanisms and amyloid plaque deposition	2018	Jorge Ghiso	CD- 10 mM phosphate buffer, pH 7.4, containing 150 mM sodium fluoride, ThT- 50 mM Tris-HCl buffer, pH 8.5
CaMKII Metaplasticity Drives Aβ Oligomer-Mediated Synaptotoxicity	2018	Daniel Choquet	F12 medium
Soluble Aβ1–42 increases the heterogeneity in synaptic vesicle pool size among synapses by suppressing intersynaptic vesicle sharing	2018	Sunghoe Chang	Ham's F-12 (phenol red-free)
A novel Aβ epitope vaccine based on bacterium-like particle against Alzheimer's disease	2018	Wei Kong	Ham's F-12 (phenol red-free)
HDL Mimetic Peptide 4F Mitigates Aβ-Induced Inhibition of ApoE Secretion and Lipidation in Primary Astrocytes and Microglia	2018	Ling LI	Ham's F-12 (phenol red-free)
Salvianolic acid B attenuates mitochondrial stress against Aβ toxicity in primary cultured mouse neurons	2018	Heng Du	Ham's F-12 (phenol red-free)
Effect of Cholesterol on Membrane Fluidity and Association of Aβ Oligomers and Subsequent Neuronal Damage: A Double-Edged Sword	2018	Luis Aguayo	MiliQ water
Effects of n-3 PUFA enriched and n-3 PUFA deficient diets in naïve and Aβ-treated female rats	2018	Lugia Trabace	MiliQ water
Estrogen deficiency exacerbates Aβ-induced memory impairment through enhancement of neuroinflammation, amyloidogenesis and NF-κB activation in ovariectomized mice	2018	Jin Tae Hong	MiliQ water
Enantiomeric Aβ peptides inhibit the fluid shear stress response of PIEZO1	2018	Philip Gottlieb	Monomer - THIP followed by DMSO then buffer as below, oligomer - 150 KCl, 10 HEPES, 1 $MgCl_2$, 1 $CaCl_2$ at pH 7.4
Astrocytic glutamatergic transporters are involved in Aβ-induced synaptic dysfunction	2018	Pingyi Xu	PBS

High-affinity interactions and signal transduction between Aβ oligomers and TREM2	2018	Todd Golde	PBS
Estrogen Receptors Are Involved in the Neuroprotective Effect of Silibinin in Aβ1–42-Treated Rats	2018	Takeshi Ikejima	PBS, pH 7.4
The diphenylpyrazole compound anle138b blocks Aβ channels and rescues disease phenotypes in a mouse model for amyloid pathology	2018	Andre Fischer	PBS, pH 7.4
The NMDA receptor antagonist Radiprodil reverses the synaptotoxic effects of different amyloid-beta (Aβ) species on long-term potentiation (LTP)	2018	Chris Parsons	Ringer's solution: 125 mM NaCl, 2.5 mM KCl, 25 mM $NaHCO_3$, 2 mM $CaCl_2$, 1 mM $MgCl_2$, 25 mM d-glucose, and 1.25 mM NaH_2PO_4, bubbled with a 95% O_2, 5% CO_2 mixture, pH of 7.3
Euxanthone Attenuates Aβ1–42-Induced Oxidative Stress and Apoptosis by Triggering Autophagy	2018	Gang Sun	Saline
Cubeben induces autophagy via PI3K-AKT-mTOR pathway to protect primary neurons against amyloid beta in Alzheimer's disease	2019	Ruijan Dong	
Neuroprotective efficacy of thymoquinone against amyloid beta-induced neurotoxicity in human induced pluripotent stem cell-derived cholinergic neurons	2019	Ikuro Suzuki	Implied cell culture medium but unclear
A catalytic antioxidant for limiting amyloid-beta peptide aggregation and reactive oxygen species generation	2019	Tim Storr	0.01M PBS pH7.4
Serotonin type 6 receptor antagonist attenuates the impairment of long-term potentiation and memory induced by Abeta	2019	Alireza Komaki	0.9% saline, pH 7.4
Exendin-4 attenuates brain mitochondrial toxicity through PI3K/Akt-dependent pathway in amyloid beta (1–42)-induced cognitive deficit rats	2019	Jaya Verma	147 mM NaCl, 2.9 mM KCl, 1.6 mM $MgCl_2$, 1.7 mM $CaCl_2$ and 2.2 mM dextrose; pH 7.4
Interactions of polyunsaturated fatty acids with amyloid peptides Aβ40 and Aβ42	2019	Praveen Rao	215mM sodium phosphate, pH 7.4
Morphological changes induced in erythrocyte by amyloid beta peptide andglucose depletion: A combined atomic force microscopy and biochemicalstudy	2019	Marco Girasole	35 mM Na_2SO_4, 90 mM NaCl, 25 mM HEPES [N-(2-hydroxyethyl)-piperazine-N1-2-ethanesulfonic acid], 1.5 mM $MgCl_2$, glucose 5 mM
Cryo-EM structure and polymorphism of Aβ amyloid fibrils purified from Alzheimer's brain tissue	2019	Mathias Jucker & Marcus Fändrich	50 mM Tris, 10 mM ethylendiaminetetraacetic acid, pH 8
Carbenoxolone Reverses the Amyloid Beta 1–42 Oligomer–Induced Oxidative Damage and Anxiety-Related Behavior in Rats	2019	Bimla Nehru	First dissolved in 100µl 100mM NaOH and further diluted in 900µl 10mM sodium phosphate
Amyloid beta-mediated KIF5A deficiency disrupts anterograde axonal mitochondrial movement	2019	Lan Guo	Ham's F-12
Senolytic therapy alleviates Aβ-associated oligodendrocyte progenitor cell senescence and cognitive deficits in an Alzheimer's disease model	2019	Mark Matteson	Incubated in DMSO for 24hrs then diluted in DMEM, 20% FBD, L-glut, 1 mM sodium pyruvate, 0.1 mM β-mercaptoethanol, 0.1 mM non-essential amino acids, and 1,000 U ml–1 leukemia inhibitory factor
9-Methylfascaplysin Is a More Potent Aβ Aggregation Inhibitor than the Marine-Derived Alkaloid, Fascaplysin, and Produces Nanomolar Neuroprotective Effects in SH-SY5Y Cells	2019	Hongze Lang	MiliQ water/ PBS
In vitro studies of the neuroprotective activities of astaxanthin and fucoxanthin against amyloid beta (Aβ1-42) toxicity and aggregation	2019	Wei Zhang	PBS
Alborixin clears amyloid-β by inducing autophagy through PTEN-mediated inhibition of the AKT pathway	2019	Ram Vishwakarma, Ajay Kumar	PBS
Mesencephalic astrocyte-derived neurotrophic factor (MANF) protects against Aβ toxicity via attenuating Aβ-induced endoplasmic reticulum stress	2019	Yuxian Shen	PBS

α-Sheet secondary structure in amyloid β-peptide drives aggregation and toxicity in Alzheimer's disease	2019	Valarie Daggett	PBS, pH 7.6
Amyloid-beta induced retrograde axonal degeneration in a mousetauopathy model	2019	Shu-Wei Sun	Saline
Hesperetin Confers Neuroprotection by Regulating Nrf2/TLR4/NF-κB Signaling in an Aβ Mouse Model	2019	Myeong Ok Kim	Saline
Trodusquemine enhances Aβ42 aggregation but suppresses its toxicity by displacing oligomers from cell membranes	2019	Christopher Dobson	ThT - 5 mM sodium phosphate, 200 µM EDTA, pH 8.0 in the presence of either 75 or 150 mM NaCl, NMR- 5mM sodium phosphate, pH 7.4
Inhibition of amyloid beta toxicity in zebrafish with a chaperone-gold nanoparticle dual strategy	2019	Siijie Lin	Undefined aqueous solution (ThT/TEM) deionized water (CD) PBS pH 7.4
Designed Cell-Penetrating Peptide Inhibitors of Amyloid-beta Aggregation and Cytotoxicity	2020	Mazin Magzoub	PBS or DMEM (no serum).
Interaction between tissue transglutaminase and amyloid-beta: Protein-protein binding versus enzymatic crosslinking	2020	Benjamin Drukarch	10 mM sodium-acetate buffer, pH 5.0 (SPR) / 70 mM NaCl, 2.5 mM $CaCl_2$, 40 mM HEPES, pH 7.5, 1 mM DTT (cross-linking)
Amyloid-Beta Peptides Trigger Aggregation of Alpha-Synuclein In Vitro	2020	Stephan Schilling	20 mM Tris/HCl, 100 mM NaCl, pH 7.0
Sinomenine inhibits amyloid beta-induced astrocyte activation and protects neurons against indirect toxicity	2020	Shiv Kumar Sharma	PBS

Supplementary Table S2: Morphometric parameters for individually 3D-reconstructed Aβ_{42} fibrils. Fibrils numbered #1-100 originate from 20mM sodium phosphate pH 8.0 assembly condition, fibrils #101-200 are from the sodium phosphate pH 7.4 assembly condition, fibrils #201-300 are from HEPES pH 7.4 assembly condition and fibrils #301-400 are from Tris pH 7.4 assembly condition. For each fibril, the following parameters are listed. N_{px}: Filament segment length in number of pixels. *L*: Contour length of the segment of the fibril on AFM height image that was traced and its surface envelope 3D-reconstructed. *h*: The average height of the fibril segment. *cod*: The mean cross-over distance of peaks on the centre fibril height profile. *dpf*: directional periodic frequency. *hnd*: twist handedness of the fibril. *csa*: The filament mean AFM tip accessible cross-sectional area of the fibril. csr: The filament mean cross-sectional radius to the helical axis. csjz: The filament cross-sectional mean second polar moment of area. The fibril numbers is the index number of each of the individual fibrils and were used throughout.

Fibril #	N_{px}	*L / nm*	*h / nm*	*cod / nm*	*hnd*	*dpf / nm^{-1}*	*csa / nm^2*	*csr / nm*	*csjz / nm^4*
**1**	509	992.20	6.35	69.35	left	-0.01442	29.77	3.00	140.84
**2**	248	482.45	7.62	60.49	left	-0.01653	41.96	3.56	290.74
**3**	391	761.74	5.91	105.24	left	-0.00950	25.89	2.80	108.56
**4**	124	240.25	6.60	75.88	left	-0.01318	27.14	2.74	171.89
**5**	1501	2929.72	4.97	44.65	left	-0.02240	16.36	2.21	48.15
**6**	266	517.59	4.97	55.14	left	-0.01813	14.08	1.97	42.76
**7**	159	308.60	4.66	50.63	left	-0.00987	12.11	1.83	28.70
**8**	167	324.25	6.11	60.94	left	-0.00821	22.72	2.54	99.15
**9**	380	740.24	7.25	124.22	left	-0.00403	38.27	3.41	234.54
**10**	320	623.07	5.74	53.16	left	-0.01881	19.53	2.23	56.55
**11**	592	1154.33	5.71	123.11	left	-0.00812	24.44	2.75	91.03
**12**	281	546.91	7.28	60.99	left	-0.01640	40.73	3.44	256.09
**13**	268	521.50	4.83	39.58	left	-0.02527	15.59	2.13	43.44
**14**	258	501.98	8.59	53.00	left	-0.01887	55.56	4.04	441.25
**15**	306	595.73	7.80	117.15	left	-0.00854	34.37	3.14	236.05
**16**	200	388.69	7.70	98.96	left	-0.00505	31.12	3.01	178.83

**17**	530	1033.27	5.78	67.88	left	-0.01473	24.64	2.61	84.04
**18**	233	453.14	4.42	67.81	left	-0.00737	9.82	1.69	16.84
**19**	419	816.42	8.51	113.74	left	-0.00879	38.45	3.29	279.31
**20**	137	265.64	6.76	39.34	right	0.01271	30.54	3.06	148.20
**21**	310	603.53	5.11	47.62	left	-0.02100	15.64	2.10	48.67
**22**	400	779.32	5.94	140.82	left	-0.00710	23.01	2.59	97.50
**23**	120	232.43	4.79	36.17	left	-0.02765	12.75	1.92	30.92
**24**	418	814.46	7.57	108.37	left	-0.00923	38.91	3.40	249.27
**25**	183	355.49	7.65	94.99	left	-0.00526	23.66	2.56	104.01
**26**	165	320.36	4.14	68.64	left	-0.01457	10.28	1.60	28.90
**27**	201	390.70	6.95	57.36	left	-0.01743	25.22	2.49	153.20
**28**	138	267.58	5.05	32.37	right	0.03090	15.82	2.20	43.75
**29**	186	361.35	7.26	85.16	left	-0.01174	31.04	2.93	220.18
**30**	773	1507.99	6.96	108.04	right	0.00926	40.77	3.31	253.62
**31**	172	334.00	5.24	67.43	left	-0.01483	15.52	2.07	47.92
**32**	309	601.58	5.02	42.88	left	-0.02332	17.42	2.26	54.73
**33**	509	992.24	7.23	105.08	left	-0.00952	30.88	3.02	173.52
**34**	311	605.48	4.87	76.20	left	-0.01312	12.60	1.92	30.72
**35**	308	599.62	8.46	57.94	left	-0.00863	38.91	3.42	264.93
**36**	526	1025.41	6.34	110.57	right	0.00904	28.34	2.94	128.52
**37**	490	955.14	6.14	131.15	left	-0.00762	24.02	2.68	106.98
**38**	347	675.82	5.18	43.63	left	-0.02292	16.46	2.10	50.78
**39**	234	455.11	6.28	98.68	left	-0.01013	20.30	2.28	95.90
**40**	215	418.04	7.57	87.96	left	-0.01137	27.09	2.68	172.74
**41**	213	414.09	10.03	74.02	left	-0.00675	58.48	4.15	558.07
**42**	197	382.81	5.99	46.93	left	-0.02131	17.82	2.23	69.34
**43**	268	521.50	6.42	139.20	left	-0.00718	31.39	3.05	182.43
**44**	324	630.87	5.83	125.63	left	-0.00796	21.45	2.51	85.03
**45**	202	392.68	4.75	31.02	left	-0.03224	15.19	2.15	40.49
**46**	173	335.95	4.91	53.28	left	-0.01877	11.40	1.73	31.55
**47**	300	583.99	4.48	88.28	left	-0.01133	11.29	1.81	23.18
**48**	262	509.79	4.36	49.65	left	-0.02014	14.41	2.03	33.10
**49**	265	515.64	6.47	72.79	left	-0.01374	27.26	2.64	116.92
**50**	503	980.47	4.93	38.05	left	-0.02628	15.74	2.21	41.91
**51**	233	453.16	5.05	40.41	left	-0.02475	15.75	2.20	43.39
**52**	439	855.48	5.05	36.70	left	-0.02725	17.46	2.32	51.52
**53**	225	437.52	5.58	46.09	left	-0.01085	16.51	2.19	55.42
**54**	805	1570.33	5.60	47.93	left	-0.02086	19.43	2.38	78.75
**55**	156	302.74	4.32	56.40	left	-0.01773	11.48	1.82	26.53
**56**	287	558.64	9.50	78.58	left	-0.01273	48.14	3.45	524.60
**57**	302	587.92	5.80	57.27	left	-0.01746	18.41	2.20	71.14
**58**	192	373.06	7.76	55.31	left	-0.01808	42.31	3.58	291.03

**59**	538	1048.85	4.75	39.05	left	-0.02561	14.37	2.07	36.76
**60**	579	1128.96	10.05	124.56	left	-0.00803	53.27	3.74	725.59
**61**	326	634.78	5.27	62.13	left	-0.01610	14.10	1.96	48.14
**62**	487	949.24	6.06	39.46	left	-0.02534	22.85	2.60	102.55
**63**	669	1304.78	4.09	24.33	right	0.04110	8.93	1.52	14.81
**64**	135	261.72	5.97	39.91	left	-0.02506	20.97	2.47	91.28
**65**	139	269.53	4.68	58.89	left	-0.01698	10.70	1.73	25.74
**66**	210	408.21	8.38	108.79	right	0.00919	36.98	3.20	258.27
**67**	217	421.88	6.55	61.17	right	0.01635	28.04	2.88	141.56
**68**	471	918.04	6.25	90.43	right	0.01106	23.73	2.50	134.45
**69**	596	1162.11	6.24	92.58	right	0.00540	20.57	2.51	70.78
**70**	241	468.76	4.97	46.27	left	-0.02161	13.72	1.94	40.53
**71**	86	166.02	6.09	15.16	left	-0.02199	27.27	2.93	120.28
**72**	430	837.92	8.61	86.35	left	-0.01158	47.54	3.77	403.25
**73**	243	472.67	5.64	50.95	left	-0.01963	19.36	2.33	71.76
**74**	160	310.55	5.80	46.39	left	-0.01078	19.21	2.39	74.06
**75**	172	333.99	5.04	41.29	left	-0.02422	16.42	2.20	51.93
**76**	259	503.93	7.55	82.52	left	-0.01212	35.25	3.14	265.10
**77**	124	240.24	5.07	52.05	left	-0.01921	15.40	2.11	47.75
**78**	457	890.63	3.55	55.13	right	0.01814	9.91	1.76	15.48
**79**	317	617.35	7.47	60.06	left	-0.01665	30.80	2.93	177.06
**80**	282	548.97	6.01	28.16	right	0.03551	25.90	2.70	99.60
**81**	175	339.93	5.32	75.86	left	-0.01318	16.60	2.15	55.25
**82**	219	425.90	7.42	53.33	left	-0.01875	44.17	3.64	289.72
**83**	264	513.81	7.43	93.68	left	-0.01068	32.05	3.05	188.42
**84**	134	259.84	6.37	70.55	left	-0.01417	18.79	2.10	104.64
**85**	162	314.82	5.37	58.84	left	-0.01699	15.21	2.06	54.10
**86**	202	392.68	4.66	42.61	left	-0.02347	13.69	1.98	38.18
**87**	128	248.11	5.96	50.84	left	-0.01967	20.62	2.48	82.71
**88**	105	203.19	5.70	55.87	left	-0.00895	14.67	2.02	47.30
**89**	217	421.99	4.82	37.15	left	-0.02692	15.84	2.20	42.50
**90**	248	482.54	8.07	39.98	left	-0.01251	47.09	3.84	360.72
**91**	233	453.24	5.46	53.72	left	-0.01861	17.75	2.27	63.60
**92**	110	212.95	5.25	41.76	left	-0.02395	14.73	1.98	53.36
**93**	194	377.05	6.31	109.11	left	-0.00917	21.66	2.49	100.14
**94**	763	1488.68	8.07	100.12	left	-0.00999	49.10	3.82	360.05
**95**	224	435.67	8.68	120.54	left	-0.00830	46.28	3.62	423.99
**96**	198	384.91	6.89	85.57	left	-0.01169	22.26	2.35	142.28
**97**	295	574.37	6.36	49.32	left	-0.02028	25.10	2.58	93.11
**98**	327	636.89	5.38	148.31	left	-0.00674	18.97	2.38	62.14
**99**	175	339.94	7.77	84.40	left	-0.01185	36.24	3.06	249.09
**100**	171	332.12	4.80	40.10	left	-0.02494	14.13	2.05	36.76

**101**	225	437.51	4.81	70.00	left	-0.01429	14.84	2.08	41.82
**102**	122	236.34	5.08	33.85	left	-0.02954	22.48	2.61	86.99
**103**	196	380.87	4.74	44.82	left	-0.02231	18.10	2.27	52.24
**104**	152	294.93	5.31	11.21	left	-0.04460	20.78	2.56	67.90
**105**	171	332.03	4.36	31.71	left	-0.03153	16.27	2.21	38.17
**106**	193	375.01	6.44	45.65	left	-0.02191	33.66	3.10	158.53
**107**	301	585.94	5.48	48.01	left	-0.02083	18.75	2.34	67.16
**108**	265	515.63	7.22	41.18	left	-0.02428	33.44	3.18	199.28
**109**	134	259.77	7.34	57.81	left	-0.00865	26.57	2.72	149.41
**110**	161	312.51	4.16	50.00	left	-0.02000	11.13	1.75	23.40
**111**	331	644.55	6.35	40.53	right	0.02467	30.96	2.98	142.72
**112**	174	337.90	5.68	51.07	left	-0.01958	18.97	2.29	65.16
**113**	233	453.15	6.88	74.80	left	-0.00668	29.93	2.92	175.89
**114**	264	513.70	4.77	76.76	left	-0.01303	18.80	2.39	52.22
**115**	185	359.34	4.36	47.66	left	-0.02098	12.53	1.90	27.46
**116**	210	408.22	4.70	14.64	left	-0.06830	16.19	2.21	43.28
**117**	232	451.18	4.22	53.71	left	-0.01862	13.19	1.95	31.52
**118**	162	314.46	6.12	81.32	left	-0.01230	32.74	3.15	170.96
**119**	202	392.60	7.63	83.30	left	-0.00400	36.90	3.33	228.44
**120**	261	507.82	7.54	75.10	left	-0.00666	34.58	3.23	201.48
**121**	153	297.02	4.43	50.39	left	-0.01984	11.80	1.83	29.82
**122**	96	185.58	5.99	35.84	left	-0.02790	18.54	2.23	77.32
**123**	185	359.39	4.91	60.40	left	-0.01656	14.79	2.02	41.13
**124**	139	269.54	4.84	22.41	right	0.04463	10.77	1.67	28.08
**125**	122	236.35	5.30	55.53	left	-0.01801	18.85	2.31	70.18
**126**	261	507.83	6.81	50.00	left	-0.02000	36.39	3.24	218.56
**127**	244	474.61	3.95	126.97	left	-0.00788	10.19	1.63	21.73
**128**	110	212.89	5.55	47.98	left	-0.02084	18.89	2.38	67.69
**129**	249	484.38	5.35	22.25	left	-0.04495	22.94	2.68	82.38
**130**	208	404.31	5.79	52.32	left	-0.01911	22.42	2.54	93.83
**131**	194	376.96	5.13	60.08	left	-0.01664	14.32	2.02	43.87
**132**	164	318.37	4.96	38.84	left	-0.01287	19.09	2.36	58.59
**133**	160	310.55	5.64	39.73	left	-0.02517	21.85	2.51	80.39
**134**	157	304.72	5.22	58.01	left	-0.00862	15.55	2.14	44.76
**135**	148	287.17	5.10	66.46	left	-0.01505	17.63	2.25	61.37
**136**	173	335.94	5.19	63.92	left	-0.01565	16.72	2.24	52.10
**137**	176	341.80	7.08	40.06	left	-0.01248	24.73	2.67	115.81
**138**	288	560.55	6.49	45.28	left	-0.01104	28.42	2.96	131.20
**139**	189	367.19	4.78	44.95	left	-0.02225	14.99	2.13	38.12
**140**	119	230.47	4.69	38.31	right	0.02610	14.33	2.07	35.84
**141**	118	228.52	5.91	37.27	left	-0.01342	25.88	2.78	117.98
**142**	246	478.53	4.95	31.13	left	-0.03212	17.67	2.31	55.50

**143**	131	253.91	7.30	70.70	left	-0.01414	24.12	2.52	137.88
**144**	298	580.24	6.48	61.30	left	-0.01631	25.46	2.72	121.93
**145**	155	300.87	7.36	72.02	left	-0.01388	31.10	2.87	220.59
**146**	291	566.57	8.86	83.32	left	-0.00600	42.48	3.48	298.28
**147**	198	384.88	6.20	44.91	left	-0.02227	27.21	2.84	131.78
**148**	297	578.32	8.31	52.47	left	-0.01906	45.30	3.38	300.73
**149**	228	443.54	6.71	41.00	left	-0.02439	41.68	3.51	260.07
**150**	226	439.62	5.05	56.51	left	-0.01769	19.45	2.34	78.00
**151**	172	334.08	5.49	62.03	left	-0.01612	19.21	2.38	67.58
**152**	237	461.06	7.56	61.15	right	0.01635	41.48	3.50	283.69
**153**	498	970.95	6.59	52.13	left	-0.01918	30.56	3.08	156.69
**154**	323	629.06	6.19	55.62	left	-0.01798	24.75	2.74	109.61
**155**	400	779.49	6.39	50.31	left	-0.00994	25.41	2.79	111.53
**156**	443	863.51	9.17	84.22	left	-0.01187	48.89	3.62	516.99
**157**	224	435.66	6.99	83.76	left	-0.01194	23.55	2.40	151.15
**158**	206	400.50	6.24	82.05	left	-0.01219	24.52	2.42	109.67
**159**	216	420.15	7.41	75.17	left	-0.00665	28.82	2.80	138.99
**160**	237	461.07	5.97	43.62	left	-0.01146	20.99	2.43	74.42
**161**	462	900.62	6.05	53.91	left	-0.01855	22.85	2.60	94.72
**162**	231	449.34	5.68	49.13	left	-0.02035	24.15	2.70	96.57
**163**	132	255.93	5.51	48.76	left	-0.02051	19.98	2.34	78.22
**164**	124	240.32	5.27	37.63	right	0.01329	14.92	1.97	50.52
**165**	270	525.55	8.25	88.93	right	0.01125	38.16	3.28	290.12
**166**	170	330.20	5.78	26.19	left	-0.01909	19.38	2.38	68.49
**167**	198	385.04	5.79	30.88	left	-0.03238	23.72	2.66	99.82
**168**	273	531.42	5.76	31.83	left	-0.01571	18.65	2.32	59.28
**169**	106	205.14	6.13	18.54	right	0.05394	30.54	3.06	139.93
**170**	122	236.39	5.49	40.28	left	-0.02482	18.82	2.37	62.70
**171**	110	212.95	5.96	49.36	left	-0.02026	25.40	2.83	103.38
**172**	94	181.85	5.23	25.79	right	0.03878	17.34	2.32	49.44
**173**	292	568.50	5.33	77.59	left	-0.01289	17.48	2.30	50.32
**174**	97	187.55	5.90	35.41	left	-0.01412	23.41	2.69	86.55
**175**	207	402.44	5.56	53.95	left	-0.01854	12.88	1.96	29.23
**176**	349	679.87	5.19	54.83	left	-0.01824	14.54	1.99	49.61
**177**	207	402.45	5.17	42.25	left	-0.02367	18.88	2.39	63.52
**178**	274	533.34	5.96	49.88	left	-0.01002	30.19	3.04	158.30
**179**	285	554.84	5.96	44.44	left	-0.01125	25.22	2.76	112.81
**180**	209	406.35	5.41	37.16	left	-0.02691	21.16	2.55	74.29
**181**	171	332.12	4.91	51.48	left	-0.01943	15.97	2.14	51.85
**182**	314	611.48	5.93	54.43	left	-0.01837	23.39	2.65	104.59
**183**	122	236.99	4.92	39.70	right	0.02519	21.72	2.59	80.17
**184**	384	748.24	6.07	48.36	left	-0.02068	28.11	2.94	134.94

**185**	162	314.55	6.45	52.32	left	-0.01911	36.10	3.14	215.90
**186**	160	310.63	7.63	60.07	left	-0.01665	40.12	3.49	290.35
**187**	277	539.22	5.89	52.31	left	-0.01912	32.80	3.17	181.34
**188**	229	445.43	5.99	48.67	left	-0.02055	25.59	2.78	111.78
**189**	420	818.58	4.78	57.15	left	-0.01750	13.62	1.89	38.22
**190**	218	423.94	5.78	41.32	left	-0.02420	26.70	2.84	129.76
**191**	361	703.30	6.29	48.60	left	-0.02058	24.50	2.73	107.88
**192**	343	668.14	4.98	50.76	left	-0.01970	14.78	2.07	42.54
**193**	173	336.03	4.95	27.47	right	0.01820	14.60	2.06	35.19
**194**	283	550.94	5.30	113.80	left	-0.00879	17.35	2.13	63.89
**195**	220	427.85	5.76	50.65	left	-0.00987	22.75	2.61	92.74
**196**	181	351.67	9.18	77.95	left	-0.01283	37.16	3.00	364.54
**197**	165	320.40	4.20	111.45	left	-0.00897	10.01	1.64	27.34
**198**	183	355.56	10.28	52.00	left	-0.00962	68.42	4.58	754.87
**199**	130	252.02	6.12	51.53	right	0.01941	21.99	2.53	98.98
**200**	272	529.44	6.60	65.80	right	0.01520	24.57	2.52	108.79
**201**	339	660.17	4.85	79.85	left	-0.01252	19.18	2.42	61.02
**202**	170	330.16	6.53	24.73	left	-0.02022	19.94	2.44	61.18
**203**	404	787.15	3.90	44.49	left	-0.02248	14.95	2.08	32.52
**204**	141	273.44	4.67	46.21	left	-0.02164	16.91	2.19	51.18
**205**	151	292.98	4.82	35.35	left	-0.02829	19.29	2.44	61.05
**206**	261	507.88	4.83	37.80	left	-0.02645	22.05	2.61	76.68
**207**	182	353.52	3.70	57.62	right	0.00868	15.54	2.16	35.91
**208**	187	363.29	4.71	42.68	left	-0.02343	19.05	2.40	55.69
**209**	255	496.11	6.02	46.41	left	-0.02155	29.24	2.99	140.90
**210**	359	699.25	4.28	91.32	right	0.01095	18.70	2.34	58.17
**211**	192	373.06	4.98	33.15	left	-0.01508	20.35	2.53	65.86
**212**	217	421.88	4.99	19.18	left	-0.02607	17.41	2.32	49.49
**213**	378	736.40	5.95	23.83	left	-0.02098	34.01	3.22	177.05
**214**	300	583.99	4.76	38.83	left	-0.01288	15.93	2.23	40.54
**215**	377	734.40	4.69	69.53	right	0.01438	16.21	2.16	40.26
**216**	286	556.65	4.60	40.29	left	-0.01241	13.88	2.05	31.11
**217**	204	396.49	4.33	40.17	left	-0.01245	13.75	2.02	29.36
**218**	428	834.02	5.10	49.19	left	-0.02033	24.59	2.65	107.58
**219**	266	517.59	5.60	41.67	right	0.01200	22.84	2.58	78.27
**220**	213	414.08	5.93	54.13	right	0.01847	24.28	2.69	101.40
**221**	261	507.82	4.94	30.20	left	-0.01656	16.86	2.30	45.64
**222**	166	322.27	4.87	29.86	right	0.03349	16.49	2.26	45.16
**223**	335	652.35	6.27	140.63	left	-0.00711	20.99	2.40	81.71
**224**	599	1167.97	6.08	139.93	left	-0.00715	22.11	2.57	77.46
**225**	391	761.75	5.06	33.19	left	-0.03013	26.11	2.85	110.12
**226**	272	529.32	4.66	39.63	left	-0.02523	19.75	2.45	64.90

**227**	765	1492.56	4.83	66.04	left	-0.00757	17.02	2.25	47.12
**228**	869	1695.42	4.19	77.54	right	0.01290	25.08	2.69	91.44
**229**	521	1015.64	4.89	51.50	right	0.01942	26.86	2.89	112.67
**230**	220	427.77	5.76	34.43	left	-0.01452	22.35	2.57	75.20
**231**	165	320.48	5.88	31.96	left	-0.01043	25.55	2.83	105.07
**232**	85	164.16	5.93	20.35	left	-0.01638	27.59	2.94	126.14
**233**	138	267.72	5.88	15.11	left	-0.03310	26.83	2.90	113.78
**234**	115	222.80	4.10	18.06	left	-0.01846	15.39	2.21	37.31
**235**	84	162.20	5.10	19.40	left	-0.01718	21.72	2.60	75.22
**236**	97	187.60	5.72	21.20	left	-0.01572	25.11	2.80	98.27
**237**	120	232.58	4.69	26.38	left	-0.01264	22.01	2.62	76.24
**238**	103	199.35	5.92	19.13	left	-0.01307	33.06	3.22	174.64
**239**	212	412.25	6.62	25.68	left	-0.01298	28.29	2.92	143.39
**240**	110	212.94	6.01	21.58	left	-0.02317	28.43	2.98	129.57
**241**	113	218.80	5.51	21.36	left	-0.02341	20.86	2.55	72.84
**242**	91	175.83	5.37	19.44	left	-0.02572	20.08	2.50	63.35
**243**	131	254.11	5.41	21.35	left	-0.02342	20.95	2.57	69.84
**244**	70	134.80	5.57	20.64	left	-0.04844	21.17	2.56	75.71
**245**	136	263.76	3.87	28.67	left	-0.01744	10.48	1.78	17.33
**246**	124	240.30	5.58	20.55	left	-0.02433	20.09	2.50	67.09
**247**	238	463.01	5.53	20.02	left	-0.02498	21.67	2.61	77.29
**248**	213	414.17	5.67	14.34	left	-0.03487	22.02	2.62	80.64
**249**	134	259.84	5.37	14.90	left	-0.06713	22.79	2.67	85.06
**250**	83	160.20	5.43	20.32	left	-0.02461	19.61	2.47	61.50
**251**	117	226.62	6.02	9.09	left	-0.10998	28.97	3.02	132.56
**252**	116	224.67	5.73	17.65	left	-0.02833	22.05	2.64	77.26
**253**	92	177.78	5.82	10.84	left	-0.02306	23.41	2.72	87.02
**254**	72	138.78	5.82	9.15	left	-0.02731	24.12	2.70	88.10
**255**	83	160.20	5.81	10.02	left	-0.01663	25.41	2.83	102.51
**256**	74	142.62	5.15	13.01	left	-0.07686	21.93	2.63	76.51
**257**	117	226.63	5.64	14.05	left	-0.01779	23.62	2.73	89.73
**258**	83	160.20	5.45	18.07	left	-0.05534	19.96	2.50	66.47
**259**	282	548.97	4.59	36.59	left	-0.02733	12.69	1.97	25.90
**260**	182	353.63	3.84	17.76	left	-0.02816	11.29	1.87	19.83
**261**	88	169.98	5.89	8.90	right	0.11236	26.22	2.86	108.31
**262**	170	330.18	5.93	16.60	left	-0.03013	26.44	2.88	111.63
**263**	55	105.50	5.59	11.33	left	-0.04413	24.05	2.75	92.15
**264**	153	296.95	6.09	19.49	left	-0.05130	29.53	3.04	138.36
**265**	99	191.45	5.95	14.78	left	-0.01691	23.93	2.73	94.44
**266**	114	220.76	5.64	15.03	left	-0.03328	21.54	2.59	74.79
**267**	179	347.75	5.48	21.83	left	-0.02291	19.82	2.48	66.56
**268**	115	222.72	5.70	13.87	left	-0.03605	24.14	2.76	92.35

**269**	143	277.41	5.69	13.65	left	-0.03664	24.42	2.77	95.01
**270**	137	265.70	5.26	29.94	left	-0.01670	19.37	2.46	60.21
**271**	246	478.65	5.92	17.75	left	-0.02817	27.16	2.92	117.67
**272**	89	171.93	6.67	39.95	left	-0.01252	25.16	2.74	122.74
**273**	190	369.63	6.08	16.64	left	-0.03004	27.06	2.92	117.41
**274**	124	240.30	5.45	23.42	left	-0.02135	20.08	2.49	65.96
**275**	252	490.36	5.35	22.04	left	-0.02269	19.17	2.43	60.78
**276**	245	476.73	5.88	14.27	left	-0.03505	24.20	2.75	93.71
**277**	141	273.51	5.80	15.20	left	-0.03289	23.92	2.74	90.89
**278**	166	322.35	5.77	22.12	left	-0.02260	22.28	2.64	83.60
**279**	177	343.84	5.69	21.61	left	-0.02314	22.20	2.63	82.19
**280**	234	455.20	6.19	15.98	left	-0.06256	29.79	3.07	141.82
**281**	163	316.49	6.15	13.58	left	-0.03681	28.67	3.00	129.83
**282**	315	613.86	4.24	52.75	left	-0.00948	8.97	1.60	14.36
**283**	185	359.47	5.82	15.76	left	-0.03172	22.47	2.65	80.49
**284**	324	631.02	5.70	14.97	left	-0.03340	24.60	2.78	95.75
**285**	187	363.38	6.33	20.42	left	-0.01225	29.92	3.08	143.74
**286**	101	195.36	6.11	15.12	left	-0.01653	25.18	2.78	106.97
**287**	157	304.84	6.05	13.86	left	-0.03607	26.33	2.86	109.74
**288**	194	377.06	5.99	13.92	left	-0.03591	25.66	2.84	105.25
**289**	106	205.14	5.82	17.35	left	-0.01921	22.27	2.64	78.38
**290**	81	156.56	6.39	22.27	left	-0.01497	27.29	2.89	127.17
**291**	91	176.71	5.74	14.13	left	-0.03538	24.11	2.76	92.72
**292**	103	199.88	5.70	20.32	left	-0.02461	23.04	2.68	88.02
**293**	98	189.60	5.34	23.39	left	-0.02138	18.18	2.36	57.90
**294**	126	244.33	4.80	21.55	left	-0.02320	14.96	2.14	36.27
**295**	106	204.85	7.52	46.76	left	-0.01069	33.20	3.15	196.39
**296**	121	233.87	4.63	36.61	left	-0.02731	14.85	2.11	39.14
**297**	271	527.70	5.70	14.01	left	-0.03570	23.99	2.74	90.28
**298**	92	176.88	5.90	14.31	left	-0.01747	24.56	2.76	94.67
**299**	93	179.28	6.05	16.16	left	-0.03095	24.45	2.76	96.86
**300**	146	283.17	4.49	22.24	left	-0.01499	12.60	1.96	26.03
**301**	205	398.46	5.06	40.63	left	-0.02462	20.15	2.46	75.85
**302**	329	640.64	3.56	54.08	right	0.01849	12.14	1.93	24.46
**303**	219	425.79	5.17	49.68	right	0.02013	21.91	2.58	82.71
**304**	281	546.88	5.19	53.75	left	-0.00930	12.89	1.94	29.47
**305**	193	375.01	5.68	32.16	left	-0.01555	19.09	2.40	65.81
**306**	134	259.77	6.83	35.13	left	-0.01423	28.04	2.90	146.15
**307**	146	283.22	4.34	15.92	right	0.06282	18.48	2.40	56.25
**308**	278	541.03	6.01	47.14	left	-0.02121	24.53	2.69	105.08
**309**	223	433.60	6.70	30.02	left	-0.03331	29.70	2.99	146.48
**310**	324	630.87	5.45	48.65	left	-0.01028	14.64	2.05	44.47

**311**	223	433.60	4.67	41.15	left	-0.02430	14.97	2.13	37.86
**312**	225	437.50	4.89	54.16	left	-0.00923	13.33	1.96	36.60
**313**	413	804.72	5.06	55.40	left	-0.01805	14.79	2.02	41.12
**314**	200	388.68	4.17	84.57	left	-0.01182	9.00	1.55	18.15
**315**	183	355.47	5.12	52.77	left	-0.01895	14.88	2.02	47.38
**316**	336	654.31	5.61	47.22	left	-0.02118	21.09	2.47	81.53
**317**	367	715.01	5.06	33.67	left	-0.01485	18.15	2.36	54.85
**318**	282	548.84	5.02	33.66	left	-0.01485	17.55	2.32	49.49
**319**	635	1238.30	4.91	41.52	left	-0.01204	17.70	2.35	51.07
**320**	470	916.03	5.67	50.07	left	-0.01997	20.08	2.40	80.82
**321**	393	765.64	5.11	54.66	left	-0.00915	13.16	1.94	34.61
**322**	661	1289.11	4.69	53.60	left	-0.01866	17.78	2.16	48.64
**323**	245	476.58	4.92	30.19	left	-0.01656	16.46	2.24	45.99
**324**	241	468.75	6.92	90.35	left	-0.01107	29.48	2.97	155.93
**325**	333	648.46	5.04	32.62	left	-0.03066	18.59	2.38	57.35
**326**	234	455.09	4.94	52.61	left	-0.01901	14.93	2.12	40.22
**327**	303	589.85	8.04	47.57	left	-0.02102	42.90	3.59	308.05
**328**	232	451.18	5.28	42.51	left	-0.01176	15.54	2.14	43.04
**329**	204	396.54	4.65	62.91	left	-0.01590	14.89	2.12	41.25
**330**	237	460.95	5.21	54.30	left	-0.01842	15.55	2.11	45.42
**331**	228	443.38	7.19	52.18	left	-0.01917	32.40	3.02	205.61
**332**	685	1336.00	4.61	73.85	left	-0.01354	11.53	1.77	22.46
**333**	214	416.02	5.72	36.56	left	-0.01368	23.32	2.69	88.30
**334**	230	447.27	4.82	37.98	left	-0.01317	16.60	2.25	45.75
**335**	248	482.48	6.02	42.75	left	-0.02339	21.10	2.38	89.03
**336**	196	380.87	5.71	41.73	left	-0.01198	18.96	2.40	65.05
**337**	538	1048.85	5.71	77.65	left	-0.01288	24.80	2.76	99.22
**338**	319	621.11	4.86	36.59	left	-0.01367	17.46	2.32	50.84
**339**	292	568.37	6.69	58.09	left	-0.00861	29.28	2.99	151.96
**340**	299	582.14	4.90	37.81	left	-0.02645	18.17	2.36	56.74
**341**	309	601.57	4.77	35.09	left	-0.01425	17.53	2.33	50.71
**342**	425	828.15	5.14	36.81	left	-0.01358	19.51	2.46	63.48
**343**	570	1111.34	5.10	45.45	left	-0.01100	16.97	2.27	49.09
**344**	351	683.60	5.06	37.42	left	-0.01336	17.71	2.34	49.42
**345**	414	806.65	4.74	41.55	left	-0.01203	15.35	2.17	37.65
**346**	498	970.73	4.24	50.51	left	-0.00990	11.99	1.89	23.40
**347**	350	681.66	3.13	162.30	left	-0.00616	6.41	1.38	7.93
**348**	277	539.07	5.48	40.97	left	-0.01221	18.06	2.34	58.89
**349**	417	812.51	4.74	35.02	left	-0.01428	14.54	2.11	36.37
**350**	298	580.09	4.82	29.43	left	-0.01699	12.59	1.89	29.12
**351**	372	724.64	5.74	40.76	left	-0.01227	16.50	2.08	49.40
**352**	391	761.77	4.32	19.63	right	0.05095	10.92	1.74	23.09

**353**	310	603.53	5.04	34.39	left	-0.01454	17.42	2.30	51.84
**354**	427	832.04	5.02	46.79	left	-0.02137	15.11	2.08	45.64
**355**	266	517.60	5.45	23.74	left	-0.01404	20.44	2.48	75.16
**356**	262	509.79	5.72	40.07	left	-0.01248	16.64	2.08	67.80
**357**	342	666.03	7.41	41.65	left	-0.02401	42.68	3.65	296.76
**358**	924	1802.76	4.70	37.01	left	-0.01351	14.74	2.12	35.53
**359**	482	939.47	4.93	41.19	left	-0.02428	17.18	2.20	47.11
**360**	374	728.53	5.54	27.73	left	-0.01202	19.16	2.42	63.49
**361**	190	369.15	6.63	41.52	left	-0.01204	25.14	2.72	123.41
**362**	303	589.85	4.76	43.98	left	-0.02274	15.20	2.14	41.18
**363**	226	439.46	5.69	23.42	left	-0.01424	23.76	2.71	93.69
**364**	400	779.31	2.97	164.99	left	-0.00606	6.23	1.36	7.00
**365**	570	1111.36	4.76	35.41	left	-0.01412	16.05	2.22	42.64
**366**	384	748.06	4.81	36.41	left	-0.02747	15.65	2.19	42.52
**367**	471	917.97	5.00	67.37	left	-0.00742	12.87	1.93	31.25
**368**	216	419.93	3.70	90.76	left	-0.01102	6.28	1.26	9.36
**369**	730	1423.87	4.26	22.33	right	0.04478	9.57	1.65	17.20
**370**	239	464.85	4.81	24.98	left	-0.02002	15.93	2.21	42.94
**371**	577	1125.05	4.70	31.33	left	-0.03191	16.53	2.24	44.95
**372**	364	709.40	5.82	40.15	left	-0.00830	19.66	2.38	76.14
**373**	713	1390.65	5.07	35.01	left	-0.02857	17.05	2.21	47.29
**374**	414	806.69	4.41	21.31	right	0.04693	14.20	2.05	34.36
**375**	601	1171.95	5.63	54.98	left	-0.01819	22.84	2.52	98.22
**376**	337	656.26	4.84	43.57	left	-0.02295	20.73	2.54	71.56
**377**	514	1001.98	4.03	93.55	left	-0.01069	17.43	2.30	48.21
**378**	224	435.56	5.10	41.84	right	0.01195	17.51	2.28	52.48
**379**	449	875.03	4.15	25.67	right	0.03895	13.54	1.98	30.32
**380**	538	1048.90	4.64	57.81	left	-0.01730	21.03	2.43	69.52
**381**	250	486.39	6.15	60.46	left	-0.00827	25.80	2.74	129.57
**382**	353	687.55	5.16	58.54	left	-0.01708	18.30	2.29	64.67
**383**	186	361.34	6.26	50.68	left	-0.01973	21.21	2.33	102.69
**384**	482	939.55	4.93	52.61	left	-0.01901	20.82	2.42	73.75
**385**	251	488.30	4.78	32.81	left	-0.03048	19.78	2.47	63.74
**386**	264	513.69	5.15	56.93	left	-0.00878	19.27	2.38	66.70
**387**	200	388.06	5.21	26.37	left	-0.01264	20.39	2.48	68.32
**388**	291	566.42	5.98	47.78	left	-0.01046	24.15	2.67	105.15
**389**	232	451.28	3.71	67.70	left	-0.01477	14.74	2.09	39.70
**390**	307	597.72	5.55	78.13	left	-0.01280	21.06	2.43	94.08
**391**	227	441.52	4.36	40.03	left	-0.01249	14.24	2.10	32.54
**392**	147	285.23	4.77	37.87	left	-0.01320	15.63	2.19	42.59
**393**	519	1011.97	4.85	58.42	right	0.01712	14.49	2.09	39.20
**394**	214	416.13	4.79	38.59	left	-0.02591	16.90	2.28	48.29

**395**	196	380.96	4.24	48.76	left	-0.01026	13.34	2.02	29.37
**396**	307	597.82	4.62	33.29	left	-0.01502	15.02	2.15	35.58
**397**	245	476.69	4.64	38.88	left	-0.01286	15.17	2.15	38.48
**398**	212	412.22	4.54	38.45	left	-0.01300	13.43	2.01	30.68
**399**	233	453.24	4.57	36.67	left	-0.02727	14.36	2.10	33.95
**400**	215	418.08	4.28	51.35	left	-0.01947	11.97	1.89	25.14

Supplementary Table S3: Morphometric parameters for individually 3D-reconstructed Aβ_{42} fibrils observed during fibril assembly. Fibrils numbered #1-38 originate from the 1-hour time point, #39-57 are from the 6 hours time-point, and 58-117 are from the 168 hours time-point during fibril assembly under the HEPES pH 7.4 assembly condition (**Supplementary Figure S7**). For each fibril, the following parameters are listed. N_{px}: Filament segment length in number of pixels. *L*: Contour length of the segment of the fibril on AFM height image that was traced and its surface envelope 3D-reconstructed. *h*: The average height of the fibril segment. *cod*: The mean cross-over distance of peaks on the centre fibril height profile. *dpf*: directional periodic frequency. *hnd*: twist handedness of the fibril. *csa*: The filament mean AFM tip accessible cross-sectional area of the fibril. *csr*: The filament mean cross-sectional radius to the helical axis. csjz: The filament cross-sectional mean second polar moment of area.

Fibril #	N_{px}	*L / nm*	*h / nm*	*cod / nm*	*hnd*	*dpf / nm*$^{-1}$	*csa / nm*^{2}	*csr / nm*	*csjz / nm*^{4}
**1**	112	216.89	4.08	32.04	left	-0.03121	11.84	1.85	23.59
**2**	93	179.74	3.62	19.39	left	-0.05157	11.00	1.76	21.01
**3**	77	148.49	3.57	22.05	left	-0.04535	12.35	1.94	23.91
**4**	84	162.16	3.51	19.73	left	-0.02535	6.81	1.39	7.26
**5**	90	173.89	4.03	24.71	right	0.04046	16.50	2.21	41.87
**6**	70	134.81	4.06	19.28	left	-0.05188	13.57	2.06	29.58
**7**	95	183.65	3.51	51.86	right	0.01928	10.97	1.81	19.25
**8**	129	250.08	4.22	41.38	right	0.01208	12.11	1.85	19.38
**9**	64	123.09	4.05	16.19	right	0.06177	14.95	2.13	35.02
**10**	145	281.33	3.76	42.68	right	0.02343	10.64	1.77	17.67
**11**	134	259.84	3.48	40.90	left	-0.02445	9.83	1.71	15.02
**12**	67	128.95	3.66	21.97	right	0.04552	10.77	1.78	19.84
**13**	86	166.06	3.92	27.86	left	-0.03590	10.79	1.79	17.61
**14**	116	224.68	3.91	72.96	right	0.01371	11.61	1.81	25.90
**15**	72	138.72	3.52	19.23	left	-0.05200	9.18	1.61	14.15
**16**	87	168.06	3.80	24.22	right	0.04128	10.13	1.69	16.91
**17**	125	242.26	3.91	20.50	right	0.02440	11.43	1.82	19.31
**18**	126	244.21	3.53	25.64	right	0.01300	9.80	1.69	15.11

**19**	78	150.43	3.58	23.41	left	-0.02136	9.74	1.69	16.73
**20**	70	134.81	3.47	24.54	left	-0.04075	12.55	1.97	26.01
**21**	79	152.40	3.57	23.28	left	-0.02148	10.74	1.76	17.85
**22**	91	175.83	3.68	32.82	right	0.01523	9.90	1.71	17.63
**23**	111	214.91	3.39	50.94	left	-0.01963	14.12	2.04	32.40
**24**	114	220.77	3.89	78.44	left	-0.01275	10.43	1.77	18.34
**25**	96	185.60	3.37	20.60	right	0.02427	9.15	1.64	13.02
**26**	148	287.26	3.07	28.97	left	-0.01726	7.14	1.48	8.13
**27**	92	177.83	3.00	28.14	left	-0.01777	7.25	1.45	8.96
**28**	67	128.99	2.75	14.17	left	-0.03529	10.56	1.81	17.74
**29**	164	318.52	3.00	25.79	left	-0.03877	7.25	1.49	8.52
**30**	130	252.09	3.17	42.52	left	-0.01176	9.13	1.66	13.84
**31**	173	336.11	3.23	39.03	right	0.01281	6.79	1.44	7.66
**32**	138	267.75	3.04	15.89	left	-0.03146	12.06	1.89	20.91
**33**	126	244.27	3.21	17.75	left	-0.02817	9.29	1.68	13.17
**34**	173	336.12	3.14	29.81	right	0.01677	8.34	1.54	11.30
**35**	302	588.27	3.07	20.15	right	0.02481	9.29	1.66	12.64
**36**	173	336.11	3.00	19.93	left	-0.02509	6.38	1.38	6.14
**37**	93	179.78	3.10	28.69	left	-0.01743	5.40	1.27	4.82
**38**	144	279.45	3.26	24.89	left	-0.02008	8.75	1.60	11.65
**39**	506	986.59	5.60	121.43	left	-0.00824	16.85	2.17	58.71
**40**	528	1029.64	6.47	80.13	left	-0.01248	28.89	2.82	140.35
**41**	377	734.57	6.17	79.12	left	-0.01264	21.28	2.44	100.27
**42**	178	345.79	6.44	56.46	left	-0.00886	19.33	2.37	69.46
**43**	153	296.96	6.25	54.08	left	-0.00925	18.53	2.24	68.47
**44**	140	271.55	6.54	56.78	left	-0.01761	21.62	2.47	94.25
**45**	401	781.48	6.46	49.48	left	-0.02021	26.23	2.67	125.49
**46**	360	701.35	6.42	67.49	left	-0.01482	24.28	2.62	109.29
**47**	118	228.65	2.68	30.57	right	0.03271	8.59	1.58	10.13
**48**	132	256.00	2.56	34.07	left	-0.01468	7.38	1.49	8.72
**49**	94	181.74	2.88	29.07	left	-0.03440	8.74	1.59	12.34
**50**	123	238.41	2.65	32.83	left	-0.03046	6.93	1.41	7.62
**51**	113	218.88	2.51	26.40	right	0.03787	7.30	1.49	8.33
**52**	150	291.17	2.66	29.09	right	0.03437	7.96	1.51	8.70
**53**	83	160.54	2.21	16.83	left	-0.02971	6.21	1.32	5.11
**54**	100	193.47	2.41	27.65	right	0.03617	8.04	1.56	10.03
**55**	82	158.29	2.82	28.18	right	0.03549	8.48	1.58	11.04
**56**	50	95.76	2.47	12.96	left	-0.03857	9.12	1.66	12.90
**57**	175	339.85	6.27	45.15	left	-0.02215	22.85	2.55	105.94
**58**	188	365.25	5.89	46.93	left	-0.01065	17.62	2.23	65.68
**59**	352	685.57	4.84	36.43	left	-0.02745	16.73	2.26	49.20
**60**	201	390.63	7.65	32.16	left	-0.03110	44.84	3.65	304.83

**61**	250	486.35	4.83	51.07	left	-0.01958	20.72	2.27	74.37
**62**	221	429.71	4.67	69.76	left	-0.01434	11.24	1.74	28.71
**63**	116	224.61	4.76	38.16	left	-0.01310	20.76	2.53	69.85
**64**	146	283.21	5.08	63.02	left	-0.00793	15.09	2.12	42.20
**65**	161	312.51	5.32	48.21	right	0.02074	22.62	2.52	92.40
**66**	131	253.91	4.90	40.23	left	-0.01243	19.09	2.44	60.42
**67**	207	402.35	5.50	53.48	right	0.01870	21.60	2.55	80.75
**68**	138	267.59	5.20	58.07	left	-0.01722	24.82	2.69	114.46
**69**	226	439.98	5.23	56.70	left	-0.01764	16.73	2.13	58.72
**70**	222	431.66	4.90	60.16	left	-0.01662	13.64	1.84	34.93
**71**	384	748.07	5.54	52.41	left	-0.01908	19.46	2.33	73.56
**72**	182	353.52	4.77	51.76	left	-0.01932	17.22	2.09	48.77
**73**	467	910.20	4.70	51.06	left	-0.01959	15.44	2.04	45.56
**74**	290	564.46	5.45	46.45	left	-0.02153	16.80	2.19	59.82
**75**	154	298.84	5.54	56.74	left	-0.01762	19.46	2.23	75.05
**76**	98	189.46	7.46	39.36	left	-0.02541	37.52	3.39	236.30
**77**	601	1171.89	4.72	65.11	left	-0.01536	14.38	1.99	41.36
**78**	164	318.37	5.02	54.15	left	-0.01847	16.05	2.12	47.47
**79**	142	275.39	7.64	13.77	right	0.07260	49.25	3.85	354.75
**80**	246	478.53	5.19	44.08	left	-0.01134	14.37	2.02	44.93
**81**	329	640.65	4.96	62.91	left	-0.01590	16.12	2.11	47.11
**82**	184	357.45	4.26	101.27	left	-0.00987	11.82	1.79	33.63
**83**	186	361.34	4.33	73.44	left	-0.01362	9.66	1.57	21.64
**84**	169	328.14	4.52	23.77	left	-0.02104	12.83	1.87	26.26
**85**	149	289.07	5.16	57.75	left	-0.01732	13.32	1.86	45.44
**86**	291	566.41	4.50	65.01	left	-0.01538	12.67	1.81	30.25
**87**	163	316.41	4.83	56.41	left	-0.01773	12.00	1.77	29.88
**88**	237	460.94	5.55	41.02	left	-0.02438	17.92	2.29	67.37
**89**	230	447.28	7.89	35.65	left	-0.02805	45.48	3.73	331.47
**90**	135	261.73	5.28	44.61	left	-0.02242	18.66	2.28	56.45
**91**	183	355.62	4.54	29.69	left	-0.01684	11.14	1.73	22.59
**92**	155	300.78	4.80	39.20	left	-0.02551	15.91	2.21	43.98
**93**	325	632.83	5.70	25.11	left	-0.01327	22.66	2.63	88.78
**94**	129	250.01	4.71	73.89	left	-0.01353	11.54	1.73	32.60
**95**	242	470.71	5.10	54.94	left	-0.00910	16.24	2.15	59.77
**96**	190	369.41	4.69	35.72	left	-0.01400	14.88	2.14	36.52
**97**	139	269.54	5.06	49.92	left	-0.02003	12.83	1.79	37.69
**98**	205	398.44	4.96	59.44	left	-0.00841	12.76	1.91	29.41
**99**	211	410.16	5.12	60.38	left	-0.01656	14.14	1.96	47.62
**100**	291	566.42	4.81	66.36	left	-0.01507	12.95	1.87	36.75
**101**	172	333.99	4.77	36.22	left	-0.02761	15.96	2.21	42.86
**102**	329	640.64	7.37	67.92	left	-0.01472	25.41	2.55	185.92

**103**	162	314.47	5.83	23.62	left	-0.01411	23.39	2.65	98.64
**104**	178	345.71	7.04	45.51	left	-0.01099	23.58	2.62	116.16
**105**	455	886.72	5.69	51.40	left	-0.01945	19.40	2.38	75.84
**106**	162	314.46	4.95	34.62	left	-0.02889	17.26	2.27	54.66
**107**	299	582.21	5.28	58.83	left	-0.01700	15.05	2.05	51.10
**108**	537	1046.89	4.03	23.34	right	0.02142	9.93	1.64	16.73
**109**	341	664.08	4.06	27.71	right	0.03609	8.78	1.54	14.47
**110**	299	582.03	5.41	51.56	left	-0.01939	17.93	2.30	66.32
**111**	447	871.11	4.73	46.83	left	-0.02135	10.50	1.62	26.42
**112**	194	376.96	5.41	49.69	left	-0.02012	17.25	2.21	61.44
**113**	247	480.48	4.85	39.86	left	-0.02509	15.87	2.19	45.34
**114**	161	312.51	7.97	56.72	left	-0.01763	44.22	3.64	324.95
**115**	158	306.65	4.58	16.99	left	-0.02943	10.70	1.78	16.92
**116**	216	419.93	4.44	17.71	left	-0.02823	9.36	1.68	13.94
**117**	151	292.97	4.98	49.53	left	-0.02019	13.11	1.91	35.92
